# Chinese bayberry exosome-like nanoparticles attenuate DSS-induced colitis via immunomodulation, barrier restoration, and microbiota remodeling

**DOI:** 10.1038/s41538-026-00880-x

**Published:** 2026-05-14

**Authors:** Mengting Wang, Zixin Shao, Zhijian Ke, Haiguang Mao, Lili Qi, Jinbo Wang

**Affiliations:** School of Biological and Chemical Engineering, NingboTech University, Ningbo, China

**Keywords:** Gastroenterology, Immunology, Microbiology

## Abstract

Inflammatory bowel disease (IBD) is a chronic intestinal disorder characterized by intestinal barrier dysfunction and immune dysregulation, with limited safe and effective therapeutic options available. Owing to their biocompatibility and bioactivity, plant exosome-like nanoparticles (PELNs) have emerged as promising natural nanotherapeutics. In this study, exosome-like nanovesicles from Chinese bayberry (*Myrica rubra*) were isolated, characterized, and termed BELNs to investigate their therapeutic potential against IBD. Purified BELNs exhibited a typical bilayer membrane structure with a 100–300 nm size. Lipidomic, proteomic, and miRNA sequencing analyses revealed that BELNs contain bioactive components, including diacylglycerols, sphingomyelin, specific proteins, miRNAs, and polyphenols like cyanidin-3-O-glucoside. In vitro experiments demonstrated that BELNs were internalized by RAW 264.7 macrophages via galactose-mediated endocytosis, reducing LPS-induced proinflammatory cytokines (TNF-α and IL-1β) and increasing anti-inflammatory IL-10. In vivo, orally administered BELNs accumulated predominantly in the murine gastrointestinal tract. In a DSS-induced IBD model, BELNs alleviated disease symptoms, preserved intestinal tissue integrity, and enhanced barrier function by upregulating tight junction protein ZO-1 and mucin MUC2. Additionally, BELNs remodeled the gut microbiota by decreasing *Escherichia-Shigella* and enriching beneficial short-chain fatty acid-producing bacteria. These findings highlight BELNs’ potential as safe and effective nanotherapeutics for treating ulcerative colitis through immunoregulation, barrier repair, and microbiota modulation.

## Introduction

Inflammatory bowel disease (IBD), which is primarily comprised of ulcerative colitis (UC) and Crohn’s disease (CD), is a chronic inflammatory disorder of the gastrointestinal tract^1^. Its pathogenesis is characterized by impaired mucosal barrier function and increased intestinal permeability. Under the influence of dysregulated intestinal flora, proinflammatory substances infiltrate the intestinal mucosa, subsequently disrupting homeostasis and triggering systemic immune responses. In recent years, the increasing incidence of IBD and its potential progression to colorectal cancer have posed significant public health challenges, particularly in China. While current therapies rely heavily on anti-inflammatory drugs or immunosuppressants, their long-term efficacy is often compromised by severe side effects. To explore safer therapeutic alternatives, this study utilized a DSS-induced experimental colitis mouse model, a widely recognized model for reliably simulating the acute inflammatory response and mucosal damage characteristic of human IBD.

Previous studies have shown that plant exosome-like nanoparticles (PELNs) can be efficiently isolated from diverse natural sources. PELNs are small nanovesicles enclosed by a lipid bilayer, trafficking highly concentrated molecular cocktails of specific proteomes, transcriptome profiles, and other active components^2^. Compared with traditional plant extracts, PELNs offer superior biocompatibility, biosafety, stability, and low immunogenicity, and the unique ability to survive harsh gastrointestinal environments to achieve targeted colonic delivery^3,4^. Owing to these various biomolecules, PELNs can target intestinal cells, macrophages, hepatocytes, and even cancer cells in the body through specific mechanisms, thereby regulating multiple physiological processes^5^. Some PELNs have been proven to reduce the risk of cancer, chronic diseases, and inflammation^6,7^. For example, oral administration of ginger-derived ELNs has been shown to have a protective effect on mice with alcohol-induced liver damage, reducing liver lipid droplet and triglyceride levels. These ELNs mainly accumulate in the liver and mesenteric lymph nodes and can induce antioxidant stress and liver-protective effects through the Nrf2 pathway^8^. Tajik et al. reported that cannabis ELNs with high cannabidiol content can effectively treat liver cancer by activating mitochondrial-dependent apoptotic signaling pathways^9^. In addition, strawberry-derived ELNs prevent oxidative stress in human mesenchymal cells, mainly through direct endocytosis^10^. A recent study revealed that tea leaf-derived ELNs can improve oleic acid-induced lipid metabolism by regulating miRNAs in HepG2 cells^11^. Despite their broad application in various diseases, to date, PELNs have been most extensively and widely investigated in the context of their protective effects against inflammation.

Emerging evidence has underscored the multitarget therapeutic potential of PELNs in mitigating symptoms in a DSS-induced IBD mouse model, primarily involving immune modulation, physical barrier restoration, and microbiota remodeling^12,13^. With respect to immune regulation, PELNs derived from *Panax* ginseng and *Houttuynia cordata* have been shown to significantly suppress proinflammatory cytokines (e.g., TNF-α and IL-6) and inhibit key inflammatory cascades, such as the NF-κB and NLRP3 signaling pathways^14,15^. Furthermore, PELNs derived from *Andrographis paniculata* can actively reprogram macrophage polarization towards the anti-inflammatory M2 phenotype^16^. In addition, broccoli-derived ELNs can alleviate colitis by inducing the formation of tolerogenic dendritic cells (DCs) via AMPK activation, thereby reducing the levels of proinflammatory IFN-γ, TNF-α, and IL-17A while increasing the level of anti-inflammatory IL-10^17^. Moreover, PELNs contribute to the maintenance of the intestinal physical barrier. For instance, miR166u-enriched PELNs from *Polygonatum sibiricum* can improve intestinal integrity by upregulating the expression of tight junction proteins (ZO-1 and Occludin) via the TLR4/AKT pathway^18^, and tangerine peel-derived ELNs protect against epithelial atrophy and increase mucin (MUC2) expression^19^. Moreover, grape-derived ELNs penetrate the intestinal mucus and stimulate the proliferation of Lgr5^+^ intestinal stem cells via Wnt signaling, thereby accelerating mucosal regeneration and preventing colitis^20,21^. Beyond direct host interactions, PELNs act as potent modulators of gut microbial ecology to alleviate IBD. Garlic-derived ELNs, specifically through the delivery of peu-miR2916-p3, can selectively increase the growth of anticolitic symbiotic bacteria such as *Bacteroides thetaiotaomicron*^22^. Similarly, ginger-derived ELNs can deliver specific miRNAs (e.g., ath-miR167a) to *Lactobacillus* to activate the AhR/IL-22 signaling axis for barrier repair^23^, whereas lemon-derived ELNs enhance the intestinal survival of the probiotics *Lactobacillus rhamnosus* GG (LGG) and *Streptococcus thermophilus* (ST-21) via the AhR pathway to effectively inhibit colitis^24^. Furthermore, PELNs from *Portulaca oleracea* L. facilitated the expansion of *Lactobacillus reuteri*, triggering the AhR-dependent reprogramming of CD4^+^ T cells into protective double-positive T cells to alleviate colonic inflammation^25^. Collectively, these studies highlight that the anti-IBD efficacy of PELNs is unlikely to be the result of a single action but rather a synergistic integration of these three processes.

However, given the vast genetic and phytochemical diversity across the plant kingdom, PELNs from different species often exhibit distinct biological signatures and functional specializations. Therefore, identifying novel PELNs sources with unique therapeutic profiles remains a priority for precision nutrition. While PELNs from various sources have shown promise, exosome-like nanovesicles from Chinese bayberry (BELNs) are compelling candidates because of their exceptional edible and medicinal value. Chinese bayberry has a well-defined history in traditional folk medicine as a dietary therapy to specifically resolve phlegm, stop chronic diarrhea, and cure dysentery, providing a more direct ethnopharmacological rationale for its use in IBD compared to general fruit sources^26^. Unlike ginger-derived ELNs, which primarily rely on gingerols, or grape-derived ELNs enriched in resveratrol, BELNs are uniquely characterized by an exceptional abundance of specific bioactive polyphenols, notably cyanidin-3-O-glucoside and myricitrin, which offer distinct phytochemical and therapeutic advantages^27^. These compounds are more potent inhibitors of the TLR4/NF-κB signaling axis in the colonic microenvironment than many other plant secondary metabolites. Chinese bayberry extract can also effectively modulate the intestinal flora of mice with antibiotic-associated diarrhea and suppress proinflammatory signaling^28^. Whether these therapeutic benefits are mediated by naturally occurring nanovesicles remains unexplored. Given the structural characteristics of PELNs, their lipid bilayer inherently facilitates the spontaneous encapsulation of potent bioactive components, such as polyphenols and specific miRNAs. BELNs have potential as integrated bionanomachines that provide superior protection and targeted delivery of bioactive components to the inflamed mucosa, thereby achieving a synergistic effect between the nanocarrier and its unique bayberry-specific cargo.

In this study, we first isolated and prepared BELNs, followed by characterization, identification, and component analysis. On this basis, by constructing a DSS-induced IBD mouse model, we explored the ability of BELNs to alleviate intestinal inflammation and preliminarily investigated the effects of BELNs on intestinal function, mainly from aspects such as intestinal morphology, intestinal physical barrier integrity, the expression of inflammatory factors, and the gut microbiota. Moreover, an LPS-induced RAW 264.7 cell inflammation model was used to further verify the internalization and immunoregulatory mechanism of BELNs in the intestine. The purpose of this study was to elucidate the ability of BELNs to alleviate intestinal inflammation and its related mechanisms, to provide new possibilities for the development of safe, efficient, and low-cost nanodrug preparations for alleviating ulcerative colitis, and to help further expand the processing and application scope of bayberries.

## Results

### Characterization of the BELNs

**Fig. 1:**
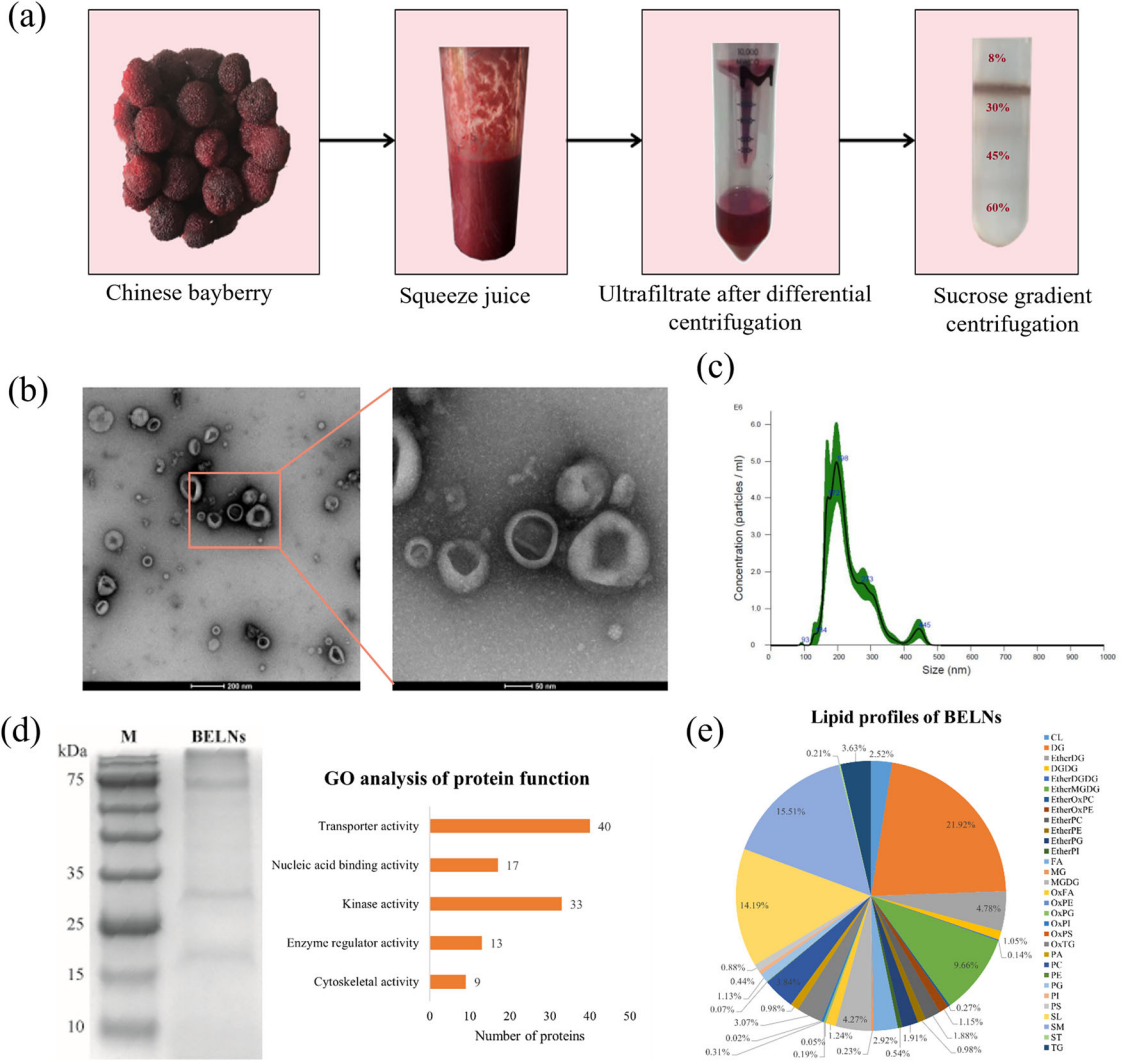
Characterization of BELNs. **a** Extraction and purification of BELNs by differential centrifugation and ultracentrifugation. **b** TEM images of BELNs. **c** NTA analysis of BELNs. **d** proteomic analysis of BELNs. **e** Lipidomic analysis of BELNs.

BELNs were extracted from Chinese bayberry fruits using differential centrifugation and purified by ultrafiltration and ultracentrifugation on a sucrose gradient to obtain samples with higher purity and more complete morphology (Fig. 1a). The morphology of the BELNs sedimented at the 8/30% interface of the sucrose gradient was observed using TEM, revealing a typical vesicular structure wrapped in a double-layered membrane shaped like a tea tray (Fig. 1b). The structure had a typical exosome-like appearance. The size of the spherical BELNs ranged from approximately 100–300 nm, which was confirmed by NTA (Fig. 1c). This finding is consistent with the morphological characteristics of PELNs derived from ginger, grape, grapefruit, and other sources, as previously reported^29^.

The protein composition profiles of the BELNs were determined using LC‒MS/MS (Supplementary Data 1). The protein population of the BELNs ranged from 25 to 35 kDa and 75 kDa. Analysis of the Gene Ontology (GO) database revealed that BELNs were associated with more than 100 types of proteins related to multiple biological processes, including transporter, nucleic acid binding, kinase, enzyme regulator, and cytoskeletal activity (Fig. 1d). The coexistence of these proteins within a lipid-rich environment characterizes BELNs as complex bioactive nanomachines rather than simple inert particles.

Lipids are important components of the vesicular phospholipid bilayer and are indispensable components of PELNs. As the main component of PELNs, the composition and proportion of lipids determine the biological distribution and transport function of PELNs in vivo^30^. Considering the double-layer membrane structure of the BELNs, the total lipid composition profiles of the BELNs were determined using LC‒MS/MS (Supplementary Data 2). Statistical analysis of the lipid contents is shown in Fig. 1e, and the major lipid components were diacylglycerols (DG, 21.92%) and ether DG (4.78%), sphingomyelin (SM, 15.51%), saccharolipids (SL, 14.19%), monogalactosyl diacylglycerols (MGDG, 4.27%) and ether MGDG (9.66%), phosphatidylcholines (PC, 3.84%), triacylglycerols (TG, 3.63%), digalactosyl diacylglycerols (DGDG, 1.05%) and ether DGDG (0.14%) (Fig. 1e). This unique lipidomic fingerprint is a key determinant of the physicochemical stability and biological behavior of BELNs. DG is a low-abundance lipid in food and has significant potential to reduce the risk factors for several chronic diseases, including obesity and postprandial hyperlipidaemia^31^. SM detected in BELNs is a known bioactive signaling molecule that can regulate cell proliferation and apoptosis^32^, and suppress proinflammatory signaling in the gut mucosa^33^. PC is an essential component of the colonic mucosal barrier^34^, whereas TG is related to lipid metabolism^35^. The high proportion of SM and PC provides a robust structural scaffold for the double-layer membrane, ensuring that the BELNs remain intact during the transition through the gastrointestinal tract. SL is hydrolyzed into metabolites throughout the gastrointestinal tract, which regulate cell growth, differentiation, and apoptosis^36^. PELNs have the advantage of being phagocytosed by animal cells without toxic side effects owing to their unique lipid outer membrane^37^. MGDG and DGDG are typically used to stabilize phospholipid liposomes during the freeze‒thaw and lyophilization processes^38^. The presence of galactolipids, such as MGDG and DGDG (14.07% combined), is a distinct characteristic of plant-derived vesicles. These saccharolipids are not merely structural components; they also provide the molecular basis for the galactose-type surface signature, which facilitates targeted uptake by macrophages, as verified in our subsequent cell experiments (Section “Uptake of BELNs by RAW 264.7 macrophages”).

The main type of RNA in PELNs is miRNA, which can enter cells and target cellular gene expression after being taken up by the organism, thereby affecting its physiological functions^39^. To assess the miRNA types in the BELNs, a miRNA sequencing analysis was conducted (Supplementary Data 3). A total of 92 known miRNAs were identified in BELNs originating from bayberry (*Morella rubra* cultivar). We found that gma-miR166a, csi-miR396, mdm-miR156a, nta-miR479a, ptc-miR6478, and ath-miR8175 were abundant in BELNs; these miRNA sequences were highly abundant and unique to Chinese bayberry fruits^40^. Among the identified miRNAs in BELNs, the miR166 family was highly abundant. Recent studies have demonstrated that plant-derived miR166 can exert potent anti-inflammatory effects through cross-kingdom regulation. For instance, Wei et al. revealed that miR166u-enriched nanoparticles can alleviate DSS-induced IBD by inhibiting the TLR4/AKT signaling pathway^18^. Furthermore, tea-derived extracellular vesicles (TEVs) enriched miR166 family members can directly target the 3’-UTR of mammalian AKT1, which decreases NF-κB levels and inhibits inflammatory responses, thereby alleviating DSS-induced intestinal inflammation in mice through cross-kingdom regulation via the delivery of plant miRNAs by TEVs to macrophages^41^. Moreover, a previous report indicated that ginger ELNs were able to mediate the gut microbiota and improve intestinal inflammation through miRNAs^42,43^, and that these exogenous plant miRNAs carried by EVs can be absorbed via the gastrointestinal tract to regulate host mRNAs^44^. These findings support our hypothesis that the high content of miR166 in BELNs can target mammalian genes involved in inflammatory signaling pathways.

Some small-molecule bioactive metabolites have also been detected in PELNs. Owing to the natural carrier function of PELNs, they can assist in the in vivo transport of small-molecule metabolites and increase their water solubility and transmembrane capacity, thereby promoting their bioactive effects in organisms^45^. Thus, the polyphenols in the BELNs were detected using HPLC in this study (Fig. S1). Cyanidin-3-O-glucoside and p-hydroxybenzoic acid are the two main polyphenol components that are present not only in the bayberry juice but also in the BELNs, along with myricitrin, quercetin, and protocatechuic acid. p-Hydroxybenzoic acid and cyanidin-3-O-glucoside were the dominant components in bayberry juice and possessed antioxidant and anti-inflammatory activities^46,47^. In particular, cyanidin-3-O-glucoside and myricitrin, characteristic bioactive components of bayberries, were found to play major physiological roles, such as alleviating the effects of antibiotic-associated diarrhea^28^. Encapsulation within the nanovesicle bilayer likely increases the bioavailability of cyanidin-3-O-glucoside and myricitrin by shielding them from the harsh acidic environment of the stomach, a protective mechanism observed in various plant-derived nanovesicles^3,48^. This encapsulation allows targeted release in the colonic microenvironment, where these polyphenols can directly interact with the damaged mucosa to exert anti-inflammatory effects^5,20^.

### Uptake of BELNs by RAW 264.7 macrophages

**Fig. 2:**
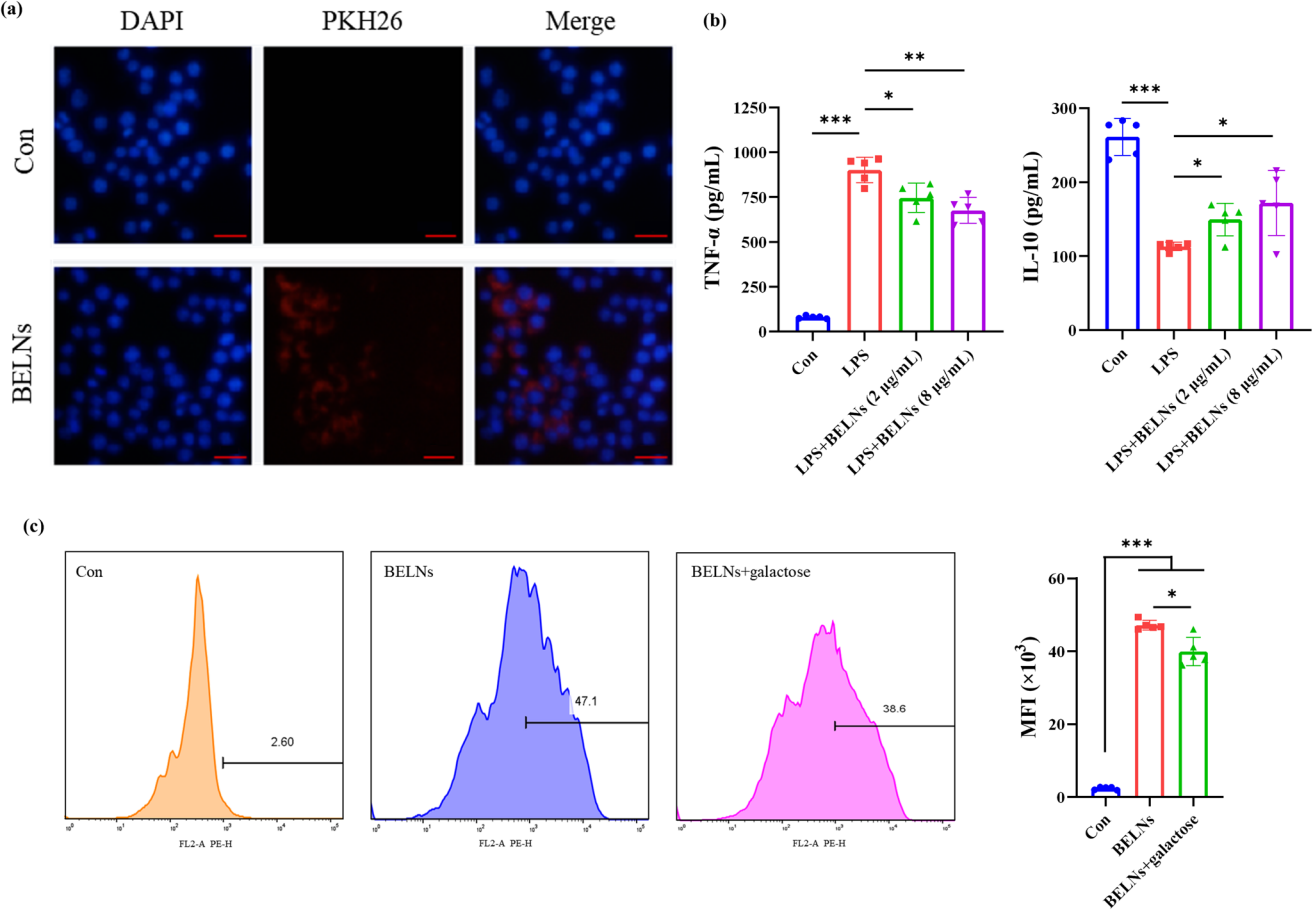
Cellular uptake and in vitro anti-inflammatory activity of BELNs in RAW 264.7 macrophages. **a** Fluorescence images of cell phagocytosis profiles of PKH26-labeled BELNs after co-incubation with RAW 264.7 macrophages for 5 h, scale bar = 10 μm. **b** Effect of BELNs on the levels of pro-inflammatory cytokine TNF-α and anti-inflammatory cytokine IL-10 in RAW 264.7 macrophages (*n* = 5). Data were analyzed using one-way ANOVA with Tukey’s post-hoc test. *p < 0.05, **p < 0.01, ***p < 0.001. **c** Flow cytometry detection of RAW 264.7 macrophages treated with PKH26-labeled BELNs in the absence and presence of galactose for 5 h (*n* = 5). Data were analyzed using one-way ANOVA with Tukey’s post-hoc test. *p < 0.05, ***p < 0.001.

Previous research has shown that plant exosome-like nanoparticles can be taken up by macrophages through endocytosis, releasing the active components within the exosomes to exert therapeutic and disease-alleviating effects^49^. To observe whether the BELNs could be successfully taken up by RAW 264.7 macrophages, the cells were incubated with PKH26-labeled BELNs over time. We first evaluated the cytotoxicity of the BELNs to RAW 264.7 macrophages (Fig. S2). BELNs at concentrations up to 8 μg/mL did not significantly affect cell viability (*p* > 0.05), indicating the excellent biosafety of the BELNs at the experimental doses. On the basis of these results, a concentration of 8 μg/mL was selected for subsequent experiments. The fluorescence images revealed no red fluorescence in the control group, whereas red fluorescence signals due to PKH26 staining were distributed on the cell outer membrane when the cells were co-incubated with BELNs for 5 h (Fig. 2a). The internalization of BELNs by RAW 264.7 macrophages was qualitatively observed.

The results of the lipid analysis described above indicated that BELNs contain a certain amount of galactose in lipid components, which is related to the natural targeting of nanoparticles to macrophages. Some studies have shown that certain nanodrugs can activate C-type galactose receptors that are overexpressed on the surface of activated macrophages under immune stress conditions, thus generating a targeted binding effect^50^. To determine the natural targeting of BELNs to RAW 264.7 macrophages, the cells were treated with PKH26-labeled BELNs in the absence or presence of free galactose for 5 h. As shown in the fluorescence staining images (Fig. S3) and flow cytometry graphs (Fig. 2c), the phagocytosis efficiency of the BELNs significantly decreased with the addition of free galactose. Here, free galactose acted as a competitive inhibitor of macrophage phagocytosis on the surface of the BELNs. These findings illustrated that galactose-type lipid membranes mediated the endocytosis of BELNs by macrophages, laying an important foundation for the bioactive functions of BELNs.

### In vitro anti-inflammatory activity of BELNs

LPS is an effective inflammatory stimulant, and macrophages activated by LPS exhibit inflammatory symptoms through the release of inflammatory cytokines and reactive oxygen species. Using an established LPS-induced inflammatory cell model, the effects of BELNs pretreatment on the cellular secretion of proinflammatory (TNF-α) and anti-inflammatory (IL-10) cytokines were evaluated (Fig. 2b). The results revealed that LPS significantly increased the TNF-α concentration and decreased the IL-10 concentration (*p* < 0.001) and that BELNs reversed these effects in a dose-dependent manner. These findings suggested that BELNs have anti-inflammatory potential, which was confirmed in animal experiments. Similarly, ELNs derived from turmeric and cabbage have been reported to prevent LPS-induced macrophage inflammation^51,52^. In turmeric ELNs, the anti-inflammatory effect is primarily attributed to the efficient delivery of curcumin and the subsequent inhibition of NF-κB-mediated M1 polarization^51^. Cabbage ELNs, on the other hand, exert their bioactivity by suppressing TLR4 signaling and reducing ROS production^52^. Consistent with these findings, we hypothesize that the anti-inflammatory potential of BELNs arises from the use of bayberry-specific polyphenols, as identified by molecular profiling (Section “Characterization of the BELNs”). Cyanidin-3-O-glucoside and myricitrin have been reported to be potent inhibitors of the TLR4/NF-κB signaling axis, which is the primary pathway through which LPS triggers proinflammatory cytokine secretion^28,47^. Moreover, the lipid bilayer of BELNs serves as a protective nanocarrier that preserves the stability of these polyphenols and facilitates their efficient cellular internalization via endocytosis^45^.

### In vivo biodistribution of DiR-labeled BELNs

**Fig. 3:**
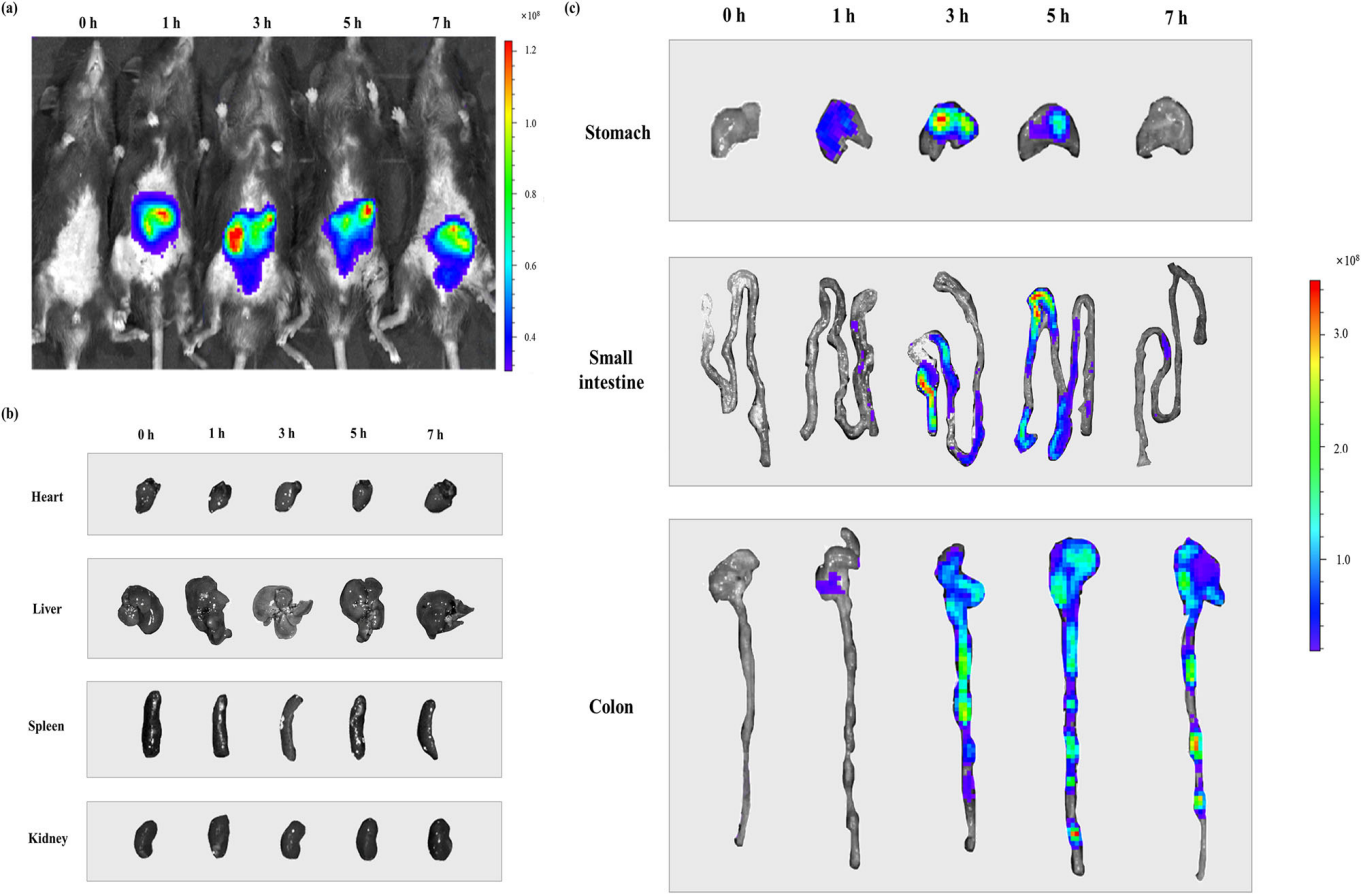
In vivo biodistribution of orally administered BELNs in mice. **a** Representative in vivo fluorescence images of the whole body of mice after oral administration of DiR-labeled BELNs. Ex vivo fluorescence images of the excised major organs, including the heart, liver, spleen, and kidneys (**b**), and the gastrointestinal tract, including the stomach, small intestine, and colon (**c**).

DiR is a lipophilic dye with satisfactory photostability for continuous excitation and is commonly used in in vivo imaging techniques^53^. In this study, DiR was used to bind BELNs and endow them with fluorescent properties. The results of the in vivo biodistribution of DiR-labeled BELNs at different time points are shown in Fig. 3. The results revealed that the BELNs could be ingested and absorbed by the mice, and the fluorescence intensity reached its maximum value at 3 h post-administration. In detail, ex vivo imaging of DiR fluorescence indicated that the BELNs were distributed mainly in the stomach, small intestine, and colon but did not penetrate the heart, liver, spleen, or kidneys. These findings suggest that BELNs can be effectively absorbed in the gastrointestinal tract, thereby exerting beneficial functions in the intestines to maintain intestinal health. Similarly, the intragastric administration of fluorescent-labeled ELNs derived from orange juice, grapefruit, mulberry bark, lemon, and ginger resulted in the predominant accumulation of ELNs in gastrointestinal organs^49^. Moreover, some ELNs, such as grapefruit-derived ELNs, have been reported to be internalized by the spleen and liver^37^.

### Protective effects of BELNs on DSS-induced IBD mice

**Fig. 4:**
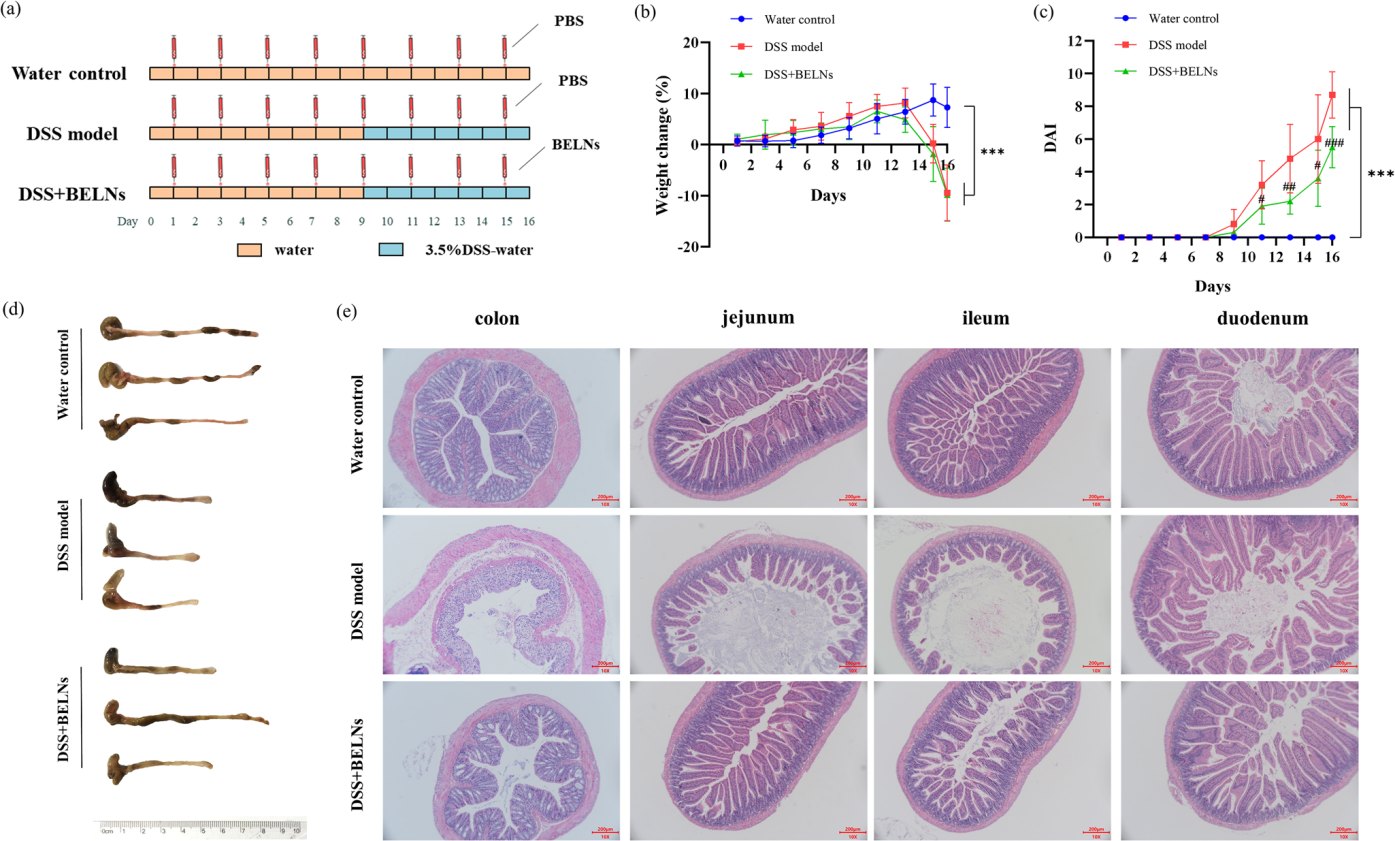
BELNs treatment ameliorates DSS-induced IBD in mice. **a** Schematic illustration of the DSS-induced experimental colitis model and BELNs treatment. **b** Monitoring the weight change of mice (*n* = 10). Data were analyzed using one-way ANOVA with Tukey’s post-hoc test. ^***^p < 0.001. **c** Evaluation of disease activity index (DAI) scores (*n* = 10). Data were analyzed using one-way ANOVA with Tukey’s post-hoc test. ^***^p < 0.001 vs. water control group, ^#^*p* < 0.05, ^##^*p* < 0.01 and ^###^*p* < 0.001 vs. DSS model group. DAI scores are the summation of the body weight loss (0- <1%, 1- 1 ~ 5%, 2- 5 ~ 10%, 3- 10 ~ 15%, 4- >15%), stool consistency (0- normal, 1/2- loose, 3/4- diarrhea), and rectal bleeding (0- normal, 1/2- occult blood, 3/4- hematochezia). **d** Intestinal morphology and length. **e** Histological analysis (hematoxylin and eosin staining, H&E) of different intestinal sections (×100 magnification), including colon, jejunum, ileum, and duodenum tissues.

In this study, a mouse model of IBD was induced by the administration of 3.5% DSS in the drinking water, and BELNs were administered (Fig. 4a). The physiological status and body weight of the mice were monitored during the experiment. The results revealed that the body weights of the mice in the DSS model group significantly decreased, but the BELNs did not obviously affect the body weights of the DSS-induced mice (Fig. 4b). Fecal traits and occult blood conditions were also monitored, and mice in both the DSS model group and the DSS + BELNs group showed diarrhea, bloody stools, and anal redness and swelling. Comprehensive analysis of the DAI score results revealed that the mice in the water control group remained healthy throughout, the DAI score of the DSS model group increased significantly, and the DAI score of the DSS + BELNs group was alleviated compared with those in the DSS model group (Fig. 4c). Colon shortening during IBD development is a critical parameter for evaluating the severity of IBD, which is attributed to mucosal damage, colonic epithelial disruption, and dehydration^54^. Changes in the colon tissues of the mice were assessed. The colon tissues of mice in the water control group were intact and had smooth surfaces, whereas those in the DSS model group showed obvious edema and congestion, and the colon length was significantly shortened. Compared with that in the DSS model group, the degree of colon tissue congestion in the DSS+BELNs group was reduced, and the colon length was slightly restored (Fig. 4d). In our study, BELNs restored the colon length from 4.80 cm in the DSS group to 5.49 cm (14.4% restoration). This restorative potency mirrors the efficacy reported for tangerine peel-derived ELNs, which restored the colon length from 5.97 cm in the DSS group to 6.82 cm (14.2% restoration) at the same dosage^19^.

Tissues from the rectal segment of the colon and the jejunum, ileum, and duodenum segments of the small intestine were collected for histopathological sectioning and H&E staining. The structural integrity of these intestinal tissues was clearly maintained in the BELNs (Fig. 4e). Specifically, in the water control group, the structural layers of the colon tissue were clear, sequentially including the muscle layer, lamina propria, and mucosal layer from the outside to the inside. Moreover, the intestinal cells were closely arranged, the goblet cells were structurally distinct, and the crypt structures were intact. However, in the DSS model group, the colon tissue significantly swells with increasing diameter, accompanied by severe tissue damage. The lamina propria was not visible in some areas, and massive numbers of cells were arranged in a disordered manner. A large number of mucosal cells were necrotic, goblet cells were depleted, and crypt structures were essentially lost. Although local swelling, partial goblet cell loss, and damage to crypt structures were observed in the DSS+BELNs group, the overall tissue structure remained relatively intact. Similarly, the small intestine tissues (jejunum, ileum, and duodenum) exhibited intact morphology, with clear microscopic structures of each intestinal wall layer in the water control group. The villi were well defined and densely arranged, with a finger-like shape in the jejunum, a slightly shorter shape in the ileum, and a leaf shape in the duodenum. Villi were moderate in length and intact, crypt structures were normal, and the mucosal epithelial tissue was complete and continuous. In contrast, the small intestine tissue in the DSS-treated group exhibited obvious swelling, with incomplete changes in the morphology of the villi and damaged structural tissue. Mucosal exfoliation occurred, numerous cavities were present within the small intestinal villi, and the crypt depth was shortened. Moreover, the small intestine tissues in the DSS + BELNs group showed slight swelling, with minimal villi exfoliation and a few cavities in the villi. Compared with that in the control group, the crypt depth in the control group was shorter, but the tissue structure remained relatively intact.

Overall, these findings indicated that DSS successfully induced experimental colitis symptoms in mice, which manifested primarily as weight loss, an increase in the DAI score, and severe disruption of the morphology and integrity of intestinal tissues. BELNs protected intestinal structural integrity and alleviated inflammatory infiltration, potentially helping to improve DSS-induced intestinal inflammatory symptoms.

### Anti-inflammatory effects of BELNs against colitis in a DSS-induced IBD mouse model

The results of in vivo biodistribution and H&E experiments indicated that BELNs may target the intestine, especially the colon, as evidenced by the accumulation of BELNs in the digestive tract after oral ingestion and the amelioration of pathological changes in colon tissue. These observations suggest that BELNs accumulation can exert anti-inflammatory effects by inducing the proliferation of intestinal stem cells and restoring the intestinal barrier in DSS-induced IBD mice^55^.

**Fig. 5:**
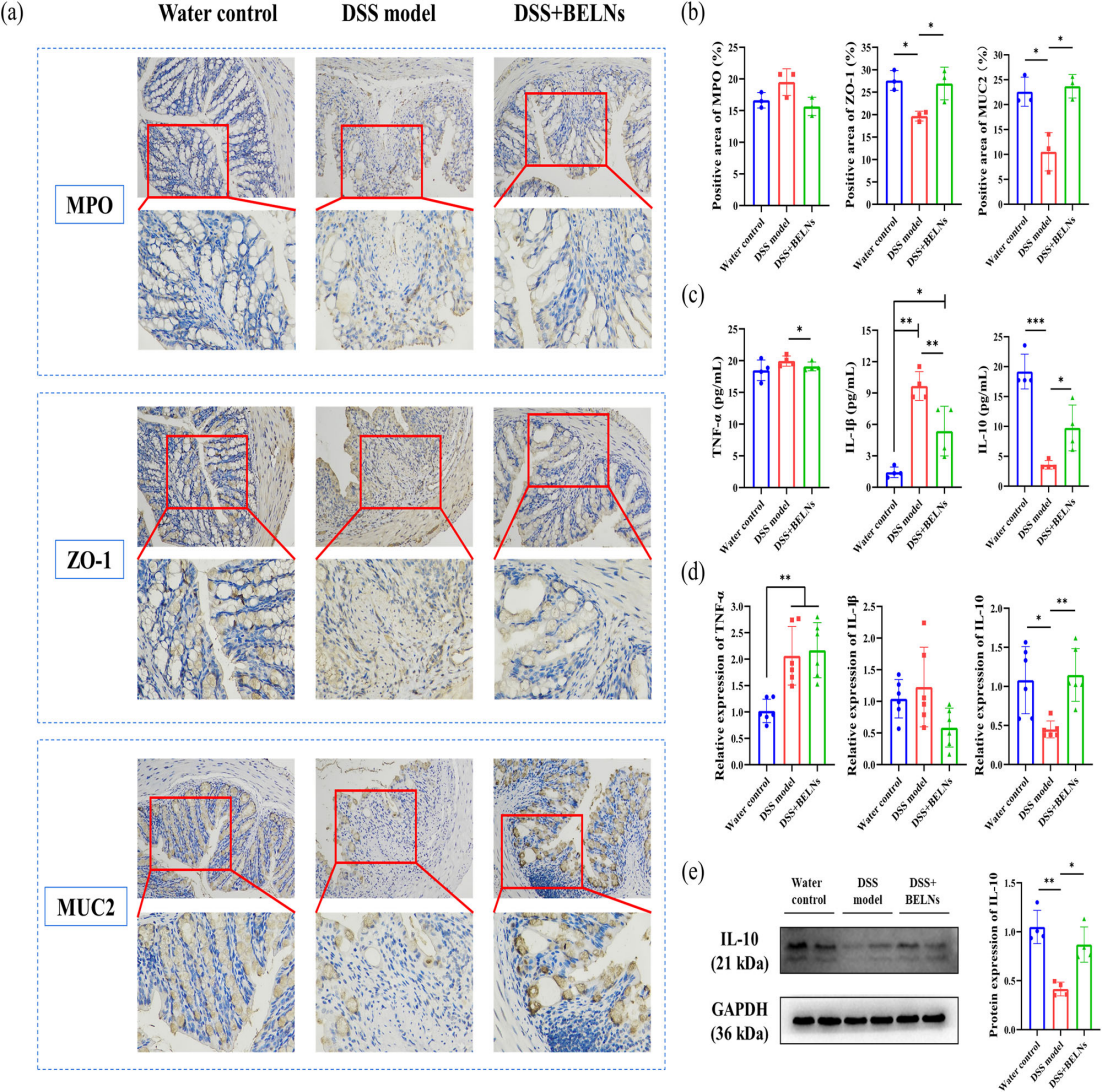
BELNs treatment enhances the expression of tight-junction proteins and anti-inflammatory factors (IL-10) in mice with DSS-induced IBD. **a** Immunohistochemical analysis of MPO, ZO-1, and MUC2 (×100 and ×200 magnification). **b** Statistics of positive areas of MPO, ZO-1, and MUC2 in colon tissues (*n* = 3). Data were analyzed using one-way ANOVA with Tukey’s post-hoc test. ^*^p < 0.05. **c** Serum levels of proinflammatory and anti-inflammatory factors (*n* = 4). Data were analyzed using one-way ANOVA with Tukey’s post-hoc test. ^*^p < 0.05, ^**^p < 0.01 and ^***^p < 0.001. **d** The RNA expression of proinflammatory and anti-inflammatory factors (*n* = 6). Data were analyzed using one-way ANOVA with Tukey’s post-hoc test. ^*^p < 0.05, and ^**^p < 0.01. **e** The western-blot bands and protein expression of IL-10 (*n* = 4). Data were analyzed using one-way ANOVA with Tukey’s post-hoc test. ^*^p < 0.05, and ^**^p < 0.01.

The distribution and expression of three marker proteins (MPO, ZO-1, and MUC2) in the colons of the mice were observed by immunohistochemistry (Fig. 5a). In terms of structural integrity, compared with those in the water control group, the intestinal mucosa of the mice in the DSS model group was severely damaged and swollen, whereas the intestinal mucosa of the mice in the DSS + BELNs group was obviously alleviated, which was consistent with the results of H&E staining. In terms of marker protein staining, compared with that in the water control group, the expression of MPO in the DSS model group increased, while the expression of ZO-1 and MUC2 decreased. The DSS + BELNs group showed a restorative effect, especially a significant increase in ZO-1 and MUC2 expression (Fig. 5b).

MPO is a bioactive enzyme stored in neutrophils and macrophages that can be released into the extracellular fluid during inflammation^56^. Therefore, MPO levels can reflect the degree of the inflammatory response. After DSS treatment, the expression of MPO in colon tissues increased, while feeding with BELNs reduced the MPO content, indicating that BELNs can decrease the infiltration of intestinal inflammatory cells. ZO-1 is a key protein in cell tight junctions and is crucial for maintaining the stability of intestinal epithelial tight junctions^57^. DSS treatment decreased the expression of ZO-1 in colon tissues, whereas BELNs increased the expression of ZO-1, suggesting that BELNs can protect the integrity of the intestinal mucosal barrier and alleviate intestinal damage. MUC2 is a key protein in the intestinal mucosa and is synthesized by goblet cells in the intestinal epithelial layer. MUC2 can filter intestinal substances and stabilize the intestinal mucosal barrier^58^. The expression of MUC2 in colon tissues was significantly reduced in the DSS model group but was significantly restored after BELNs administration, indicating that BELNs can promote the repair of damaged mucosa, improve intestinal barrier function, and reduce inflammation. Similarly, *Polygonatum sibiricum*-derived ELNs significantly ameliorated disease severity, restored colonic histological architecture, and enhanced intestinal barrier integrity by upregulating the expression of tight junction proteins (ZO-1 and Occludin) and mucin (MUC2) in a DSS-induced IBD model^18^.

Furthermore, the levels of inflammatory cytokines, including the proinflammatory factors TNF-α and IL-1β, as well as the anti-inflammatory factor IL-10, were detected in mouse serum (Fig. 5c). The results revealed that compared with that in the water control group, the content of IL-1β in the DSS model group significantly increased (*p* < 0.01), whereas the content of IL-10 significantly decreased (*p* < 0.001). Compared with those in the DSS model group, the levels of TNF-α (*p* < 0.05) and IL-1β (*p* < 0.01) in the DSS + BELNs group were reduced, with suppression rates comparable to those of tangerine peel-derived ELNs^19^. Furthermore, BELNs significantly increased serum IL-10 levels (*p* < 0.05) and upregulated colonic expression at both the mRNA and protein levels (Fig. 5d, e). Cytokine IL-10 signaling is a central orchestrator for preserving gastrointestinal homeostasis, primarily by restraining colonic inflammation through myeloid-mediated responses and the promotion of anti-inflammatory lamina propria macrophages (LPMfs)^59^. In the present study, the marked upregulation of local and systemic IL-10 levels following BELNs treatment indicated a potent immunomodulatory capacity that is on par with the recently reported high-efficiency *Portulaca*-derived ELNs^25^. By significantly enhancing this anti-inflammatory axis, BELNs effectively reconfigure the mucosal microenvironment, shifting it from a state of chronic inflammation towards stable homeostatic recovery.

### BELNs treatment reshapes the gut microbiota in a DSS-induced IBD mouse model

**Fig. 6:**
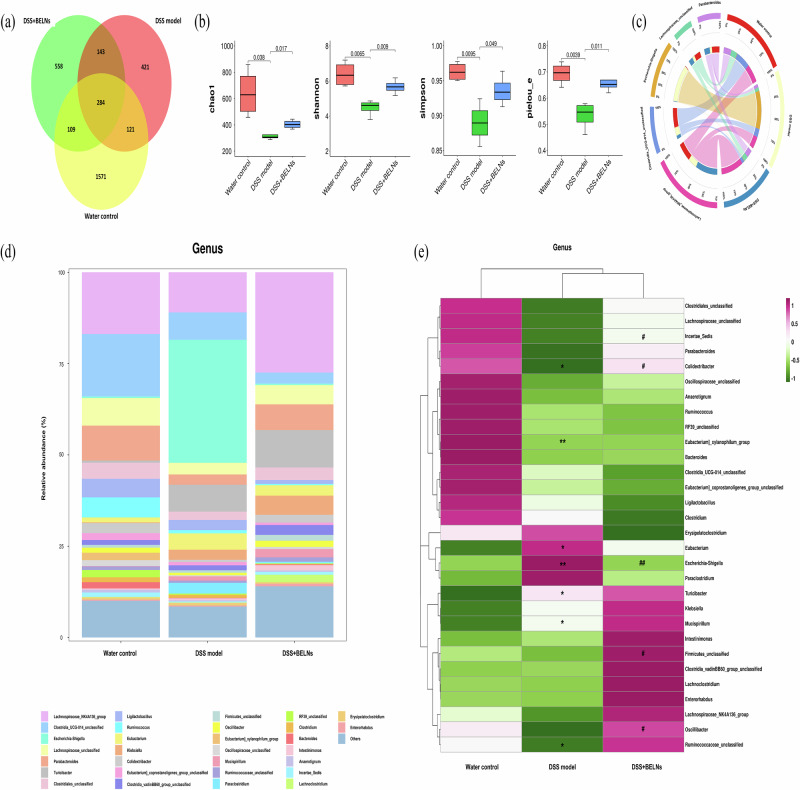
BELNs treatment modulates gut microbiota in mice with DSS-induced IBD. **a** Venn diagram of OTUs distribution. **b** Alpha diversity was compared using the Kruskal–Wallis test. **c** Circos plot of the top 5 dominant microbial groups (genus level). **d** Gut microbiota composition and distribution (genus level). **e** A genus-level community heatmap. Data are pooled in one independent experiment with *n* = 5 mice per group.

As previously discussed, PELNs can accumulate in the colon, which is home to trillions of bacteria, and their presence plays a critical role in the gut microbiota^60,61^. To determine whether BELNs treatment changed the gut microbiota in DSS mice, the fecal bacteria were analyzed using 16S rDNA sequencing technology. A Venn diagram was generated to show the differences in community composition among the three experimental groups. As shown in Fig. 6a, 284 OTUs coexisted in all groups; 1571 OTUs appeared in the water control group, 421 in the DSS model group, and 558 in the DSS+BELNs group. The α-diversity and β-diversity among the three groups were also evaluated to determine the richness and diversity of the gut microbiota. Different α-diversity indices, including the Chao, Shannon, Simpson, and Pielou_e indices, tended to decrease α diversity in the DSS group, whereas this phenomenon was reversed by treatment with BELNs (Fig. 6b). The differences in principal component analysis (PCA) among the three groups and the PCoA based on the Bray–Curtis distance among the three groups are presented in Fig. S4. The DSS model groups and water control group were clearly separated, whereas the DSS + BELNs group and water control group exhibited overlapping distributions. These findings indicate that the bacterial composition was greatly altered after DSS treatment, but the bacterial composition in the DSS+BELNs group was closer to that in the control group.

Changes in the composition of the gut microbiota at the phylum and genus levels were analyzed. *Firmicutes*, *Proteobacteria*, *Bacteroidota*, *Deferribacterota*, and *Actinobacteriota* were the 5 most abundant phyla (Fig. S5a). Compared with those in the water control group, the abundance of *Proteobacteria* significantly increased, and the abundance of *Firmicutes* significantly decreased in the DSS model group, but these changes were reversed after BELNs treatment (Fig. S5b, c). At the genus level, *Lachnospiraceae_NK4A136_group*, *Clostridia_UCG-014*, *Escherichia-Shigella*, *Lachnospiraceae*, and *Parabacteroides* were relatively enriched in the three groups (Fig. 6c). Specifically, the control group was composed mainly of *Lachnospiraceae_NK4A136_group*, *Clostridia_UCG-014*, *Lachnospiraceae*, *Parabacteroides*, *Clostridiales*, *Ligilactobacillus* and *Ruminococcus*, whereas the DSS group was composed mainly of *Escherichia-Shigella*, *Lachnospiraceae_NK4A136_group*, *Clostridia_UCG-014*, *Turicibacter* and *Eubacterium*, and the DSS + BELNs group was composed mainly of *Lachnospiraceae_NK4A136_group*, *Clostridia_UCG-014*, *Lachnospiraceae*, *Parabacteroides*, *Turicibacter*, *Clostridiales*, *Eubacterium, Klebsiella*, *Clostridiales_vadinBB6Q_group*, *Mucispirillum* and *Lachnoclostridium* (Fig. 6d). On the basis of the abundance of OTUs at the genus level, the top 30 most abundant bacteria were clustered hierarchically, and a comparative heatmap was constructed to analyze the gut microbiota among the three groups (Fig. 6e). Compared with those in the water control group, the abundances of *Escherichia-Shigella*, *Eubacterium*, *Turicibacter* and *Mucispirillum* significantly increased, whereas the abundances of *Colidextribacter*, *Eubacterium_xylanophilum_group*, and *Ruminococcaceae* significantly decreased in the DSS model group. On the other hand, compared with those in the DSS model group, the abundances of *Escherichia-Shigella* significantly decreased, whereas the abundances of *Incertae_Sedis*, *Colidextribacter*, *Firmicutes*, and *Oscillibacter* significantly increased in the DSS+BELNs group. Notably, overabundant pathogen species such as *Escherichia-Shigella* were strongly associated with inflammation-related traits in mice with colitis, which is consistent with a previous report^62^, whereas *Colidextribacter* may be related to the remission of colitis mediated by BELNs. Studies on a DSS-induced IBD mouse model have revealed that fucoidan can effectively inhibit colonic inflammation and repair the intestinal barrier. The underlying mechanism may be related to an increase in the relative abundance of *Lachnospiraceae* (including *Colidextribacter*), which in turn increases the production of short-chain fatty acids, particularly butyrate^63^. *Clostridiales_Family_XIV._Incertae_Sedis* and *Oscillibacter* may be beneficial bacteria for intestinal health, and it is thought that these bacteria exert potential anti-inflammatory and protective effects by producing SCFAs and maintaining the balance of the microbiota^64–66^.

**Fig. 7:**
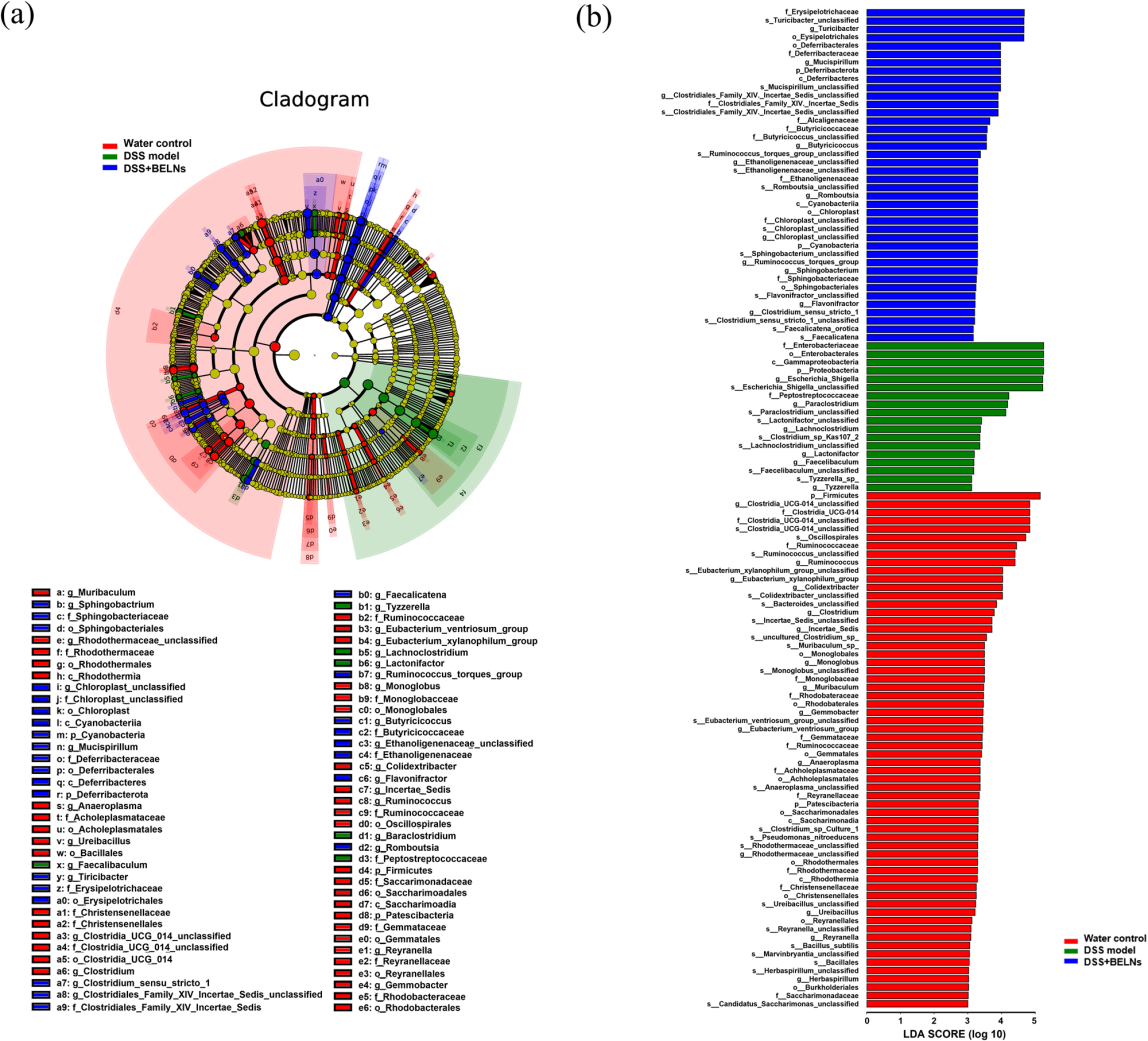
LEfSe analysis identifies specific gut microbial biomarkers modulated by BELNs treatment. **a** Taxonomic cladogram illustrating the phylogenetic distribution of significantly different bacterial lineages among the water control, DSS model, and DSS+BELNs groups. **b** Histogram of the linear discriminant analysis (LDA) scores revealing the differentially abundant taxa across the three groups. A logarithmic LDA score threshold of >3.0 was used to identify significant biomarkers.

LDA effect size (LEfSe) can be used to analyze biomarkers with significant differences in abundance across all taxonomic levels between different groups (Fig. 7). In the cladogram (Fig. 7a), the different circular layers radiating from the inside to the outside represent the seven taxonomic ranks. Each node represents a taxon at the corresponding level, and the larger the node is, the greater the abundance of that taxon. Among them, nodes colored red, blue, or green indicate that the taxon significantly differs between the comparison groups, and the abundance of this taxon is greater in the corresponding group^67^. The distribution histogram (Fig. 7b) mainly displays the significantly different species with an LDA score greater than the preset value (3.0), which are the biomarkers with significant differences. The larger the LDA score is, the greater the impact of the significantly different species. According to the results of the analysis, *Enterobacteriaceae* (from the family to the order), *Proteobacteria* (from the phylum to the class), *Escherichia-Shigella* (from the genus to the species), *Peptostreptococcaceae* (family), and *Paraclostridium* (from the genus to the species) were the key types of bacteria that led to intestinal microbiota imbalance in the DSS model group. However, *Erysipelotrichaceae* (from the family to the order), *Turicibacter* (from the genus to the species), *Deferribacterales* (from the family to the order), *Mucispirillum* (from the genus to the species), *Clostridiales_Family_XIV._Incertae_Sedis* (from the family to the species), *Alcaligenaceae* (family), *Butyricicoccaceae* (from the family to the species), and *Ruminococcus_torques_group* (from the genus to the species) were enriched in the DSS + BELNs groups. Among them, *Erysipelotrichaceae* and *Turicibacter* had the highest LDA scores and are likely the key gut microbes involved in the BELNs treatment. Overall, LEfSe analysis revealed significant differences between the three groups at multiple taxonomic levels, and these differential species may be associated with disease progression against colitis.

To further elucidate the functional consequences of BELNs-mediated microbial remodeling, we performed high-resolution PICRUSt2 functional prediction analysis (Fig. S6 and Supplementary Data 4). As shown in Fig. S6a, BELNs treatment significantly reversed the DSS-induced suppression of the fatty acid biosynthesis pathway and amino acid-related enzymes. The restoration of fatty acid biosynthetic potential is a critical metabolic driver, as it directly correlates with the increased production of SCFAs. At a more granular level, the upregulation of specific enzymes such as β-xylosidase and UDP-N-acetylglucosamine enolpyruvyl transferase in the BELNs group (Fig. S6b) suggests an enhanced capacity for complex carbohydrate degradation and bacterial cell wall maintenance. The predicted enrichment of β-xylosidase, an enzyme essential for the hydrolysis of complex plant hemicelluloses into fermentable sugars, suggests an enhanced microbial capacity for the production of SCFAs. These SCFAs, particularly butyrate, serve as the primary metabolic fuel for colonocytes, providing the highly energetic substrates required for the biosynthesis and structural assembly of the intestinal barrier components ZO-1 and MUC2, which are energy-dependent barrier components^18,61^. Furthermore, the normalization of diaminopimelate decarboxylase (the terminal enzyme in lysine biosynthesis) and pathways related to cysteine and methionine metabolism reflects a shift towards an anti-inflammatory metabolic milieu. These microbe-derived amino acids and their submetabolites serve as critical signaling molecules; for instance, lysine and its derivatives can act as AhR ligands or modulate Treg differentiation, whereas sulfur-containing amino acids are essential for maintaining redox homeostasis and suppressing oxidative stress-induced TLR4/NF-κB activation. This metabolic recalibration aligns with our observations of IL-10-mediated immune resolution and reinforces the synergistic role of the gut microbiota in BELNs-treated mice. Although direct measurements of fecal metabolites and FMT experiments were not performed, the high degree of consistency between our taxonomic findings and the predicted metabolic enrichment (Supplementary Data 4) provides a robust mechanistic framework for the anti-IBD role of BELNs. Future studies involving targeted metabolomics and FMT will be prioritized to provide direct causal evidence of these interactions. Taken together, these findings revealed that BELNs treatment significantly altered the diversity and composition of the gut microflora, which partially reversed the imbalance in the gut microbiota in DSS-induced IBD mice. Our findings demonstrate the potential of BELNs in gut microbiome modulation and IBD treatment. Notably, this ability to actively reshape the microbial landscape is a distinct therapeutic advantage over conventional anti-inflammatory drugs such as 5-aminosalicylic acid (5-ASA). While 5-ASA primarily targets mucosal inflammatory mediators to provide symptomatic relief^14^, BELNs orchestrate a more comprehensive remodeling of the gut microbial ecosystem, addressing the underlying dysbiosis that often triggers IBD relapse. This multidimensional approach positions BELNs as a more holistic natural intervention strategy for long-term intestinal homeostasis.

## Discussion

In this study, exosome-like nanoparticles from Chinese bayberry (BELNs) were successfully isolated, characterized, and demonstrated to be a sophisticated, multicomponent natural delivery system capable of significantly alleviating DSS-induced inflammatory bowel disease (IBD) in mice. Unlike conventional single-target anti-inflammatory drugs such as 5-aminosalicylic acid (5-ASA), which primarily provide symptomatic relief, BELNs function as integrated bio-nanomachines. They orchestrate a comprehensive and synergistic cascade of therapeutic events, encompassing immunomodulation, intestinal barrier restoration, and gut microbiota remodeling, to shift the dysbiotic inflammatory environment towards stable homeostatic recovery.

The therapeutic efficacy of BELNs is fundamentally rooted in their unique physicochemical architecture and bioactive components. At the structural level, the high enrichment of SM and PC provides a robust lipid scaffold that preserves double-layer membrane integrity during harsh gastric transit. Our in vivo biodistribution analysis confirmed that BELNs successfully survived the digestive environment and accumulated predominantly in the murine gastrointestinal tract, particularly the colon, without penetrating major off-target organs like the heart or liver. This localized accumulation is a significant therapeutic advantage, as it ensures high local concentrations of bioactive components while minimizing potential systemic toxicity. This allows the shielding of sensitive polyphenols, such as cyanidin-3-O-glucoside and myricitrin, from degradation to ensure colonic delivery while simultaneously enriching miR166 family members with cross-kingdom regulatory potential. This synergistic integration of targeting lipids, protected polyphenols, and regulatory miRNAs enables BELNs to function as an integrated bio-nanomachine that orchestrates a cascade of therapeutic events.

Mechanistically, the presence of galactolipids (MGDG and DGDG) constitutes a specific galactose-type surface signature that facilitates targeted cellular internalization by mucosal macrophages via galactose-mediated endocytosis. By demonstrating the efficient uptake of BELNs by RAW 264.7 macrophages, this study identifies the primary cellular vehicle through which BELNs suppress the inflammatory milieu. Once internalized, the highly concentrated molecular cocktails within BELNs exert potent immunomodulatory effects. Consistent with findings from turmeric- and cabbage-derived ELNs, which suppress NF-κB and TLR4 signaling, BELN-encapsulated polyphenols (such as cyanidin-3-O-glucoside and myricitrin) directly inhibit the secretion of proinflammatory cytokines (TNF-α and IL-1β)^28^. Crucially, the encapsulation of these polyphenols within the BELNs lipid bilayer likely provides a protective shield against harsh gastric acid, thereby enhancing their bioavailability and ensuring their targeted delivery to the inflamed colonic mucosa. Once internalized by mucosal macrophages, these polyphenols may directly inhibit proinflammatory cytokine secretion while simultaneously fostering a microenvironment conducive to IL-10-mediated immune resolution. This effect provides a plausible explanation for the significant reduction in systemic inflammation and the restorative immune response observed in the BELNs-treated mice^46,47^.

Beyond immune regulation, BELNs significantly contribute to the maintenance and restoration of the physical intestinal barrier. IBD pathogenesis is characterized by impaired mucosal integrity, which triggers systemic immune responses. Our findings revealed that BELN administration prevented colon shortening, reduced myeloperoxidase (MPO) infiltration, and upregulated the expression of critical tight junction proteins (ZO-1) and mucin (MUC2). This restorative potency is likely augmented by cross-kingdom regulation mediated by plant-derived miRNAs. BELNs are highly abundant in the miR166 family, which has been shown in previous studies involving *Polygonatum sibiricum* and tea-derived nanovesicles to exert potent anti-inflammatory effects and stabilize the epithelial layer by targeting mammalian inflammatory signaling axes^18^.

Crucially, the restoration of intestinal barrier function is intimately linked to the extensive remodeling of the gut microbiota, creating a vital regulatory feedback loop. BELNs treatment successfully reversed DSS-induced dysbiosis by restricting the expansion of proinflammatory pathogens such as *Escherichia-Shigella* while enriching beneficial short-chain fatty acid (SCFA)-producing bacteria, notably *Colidextribacter*, and *Oscillibacter*. This reshaping is likely driven by a dual mechanism. First, the intact polyphenols reaching the colon exert a prebiotic-like effect and are biotransformed by local microbiota into smaller, highly bioactive phenolic metabolites. On the one hand, these polyphenols exert a prebiotic-like effect by selectively promoting the growth of beneficial bacteria. Studies have shown that cyanidin-3-O-glucoside can specifically increase the abundance of SCFA-producing genera^28^, which aligns with our findings of increased *Colidextribacter* and *Oscillibacter* abundance. On the other hand, the vast majority of BELNs-encapsulated polyphenols reach the colon intact because of the protection of the lipid bilayer, where they are biotransformed by the local microbiota into smaller phenolic metabolites. These microbial metabolites often possess superior anti-inflammatory and antioxidant activities compared with their parent compounds, further stabilizing the intestinal microenvironment and inhibiting the overgrowth of pH-sensitive pathogens such as *Escherichia-Shigella*^46,47^. Second, exogenous plant miRNAs delivered by BELNs can be selectively taken up by gut bacteria, modulating microbial gene expression and metabolic activities. Recent studies have highlighted the role of plant-derived miRNAs in cross-kingdom communication, demonstrating that they can be selectively taken up by specific gut bacteria (such as *Lactobacillus*) in a sequence-dependent manner^23^. These exogenous miRNAs can subsequently modulate bacterial gene expression, influencing microbial growth and metabolic activities, including the production of SCFAs and aryl hydrocarbon receptor (AhR) ligands^43^. Therefore, the high abundance of miR166a and other bioactive miRNAs in BELNs likely serves as a potential regulatory bridge that favors the expansion of SCFA-producing bacteria such as *Colidextribacter*. However, this hypothesis remains untested and represents a limitation of the current work. Owing to the complexity of the intestinal environment, the direct regulatory role of specific BELNs-miRNAs on gut bacteria requires further verification through targeted miRNA mimic experiments or fecal microbiota transplantation (FMT) in future studies^42,44^.

Functional predictions (PICRUSt2) further corroborated this microbiota-barrier axis, revealing an enhanced microbial capacity for SCFA production and an anti-inflammatory shift in amino acid metabolism. SCFAs, particularly butyrate, serve as the primary metabolic fuel for colonocytes and are essential for the energy-dependent biosynthesis and structural assembly of ZO-1 and MUC2^61,63^. Consequently, the restored physical barrier and robust mucus layer provide a stable ecological niche for the colonization of these beneficial microbes, thereby solidifying long-term intestinal homeostasis.

While our in vivo data confirmed the restoration of the intestinal microenvironment, the intestinal barrier is a complex multicellular system. The direct interaction between BELNs and intestinal epithelial cells (IECs) remains unexplored in vitro. Future studies utilizing intestinal epithelial models, such as Caco-2 cells or HT-29 cells, are warranted to elucidate whether BELNs exert direct cytoprotective effects independent of their immunomodulatory capacity^68^. Additionally, owing to the complexity of the gut ecosystem, the precise regulatory roles of specific BELNs-derived miRNAs and polyphenols on bacterial metabolism require further verification through targeted metabolomics and FMT. Collectively, these findings highlight BELNs as safe, highly biocompatible, and multi-targeted natural nanotherapeutics, offering a promising holistic intervention strategy for ulcerative colitis and expanding the functional application of Chinese bayberries. Future studies will focus on elucidating the specific molecular mechanisms (e.g., miRNA-mediated regulation and metabolic pathways) and optimizing BELNs delivery efficiency through exosome engineering for clinical translation^5,69^.

## Methods

### Isolation and purification of BELNs

Chinese bayberries (*Myrica rubra*, “Biqi” cultivar) were harvested in June from an orchard in Ningbo, Zhejiang Province, China. Fruits were selected for uniform maturity and size. Chinese bayberries were subsequently washed and drained, after which the pits were removed. The juice was extracted by homogenization in a blender and centrifuged sequentially at 4 °C under the following conditions: 1000 × *g* for 10 min, 2000 × *g* for 20 min, 4000 × *g* for 30 min, and 10,000 × *g* for 1 h. The solution was filtered through a 0.22 μm membrane. The supernatant was ultracentrifuged (100,000 × *g*, 2 h) using an Optima™ XPN ultracentrifuge (Beckman Coulter, USA). The precipitate was subsequently washed and resuspended in PBS (pH 7.2) and subsequently purified on a sucrose gradient (8, 30, 45, and 60% sucrose in 20 mM Tris-HCl, pH 7.2) at 100,000 × *g* for 2 h. The BELNs in the 8/30% layer were subsequently harvested.

### Morphology and nanoparticle size of BELNs

The BELNs sample was dropped onto an ultrathin copper grid, mixed with a 1% uranyl acetate solution for negative staining, and then placed under a Talos 120 kV cryo-transmission electron microscope (TEM) (Thermo Fisher Scientific, USA) to observe the morphology. The size of the BELNs was analyzed using a NanoSight NS500 nanoparticle tracking analyser (NTA) (Malvern, UK).

### Lipids, proteomics, and microRNA sequencing analysis of BELNs

For lipid analysis, the BELN samples were analyzed using LC‒MS/MS by BiotechPack Scientific Co., Ltd. (Beijing, China). In brief, lipids from BELNs were extracted using methanol, methyl tert-butyl ether, acetonitrile, and isopropanol and investigated using an Ultimate 3000 HPLC (Thermo Fisher Scientific, USA) equipped with a triple TOF5600 mass spectrometer (Applied Biosystems SCIEXTM, USA).

For proteomic analysis, proteins from the BELNs were separated by SDS‒PAGE, and the gels stained with Coomassie Brilliant Blue were subjected to LC‒MS/MS identification by Oebiotech Co., Ltd. (Shanghai, China). Briefly, the resulting protein bands were enzymatically digested and desalted, and then detected using an Ultimate 3000 HPLC (Thermo Fisher Scientific, USA) equipped with a Q Exactive Plus mass spectrometer (Thermo Fisher Scientific, USA).

For the miRNA sequencing analysis, total RNA from the BELNs was isolated and purified, after which a library was generated using a TruSeq Small RNA Sample Prep Kit (Illumina, San Diego, USA), and the miRNA was sequenced using an Illumina HiSeq2500 by LC-Bio Technologies Co., Ltd. (Hangzhou, China).

### Polyphenol analysis of BELNs by HPLC

BELNs were dissolved in methanol and filtered through a 0.22 μm membrane filter. The polyphenol compositions of BELNs were analyzed using an HPLC (Waters e2695, USA). The specific detection conditions are as follows: chromatographic column: Agilent ZORBAX-SB C18 (100 × 4.6 mm^2^ i.d., 1.8 μm), flow rate: 0.7 mL/min, column temperature: 30 °C, injection volume: 10 µL, detection wavelength: 280 nm for phenolic acids and 520 nm for anthocyanins, mobile phase A: 0.5% formic acid-aqueous solution, mobile phase B: pure methanol, gradient elution: 0–5 min, 90–85% A; 5–6 min, 85–80% A; 6–10 min, 80% A; 10–11 min, 80–75% A; 11–15 min, 75–60% A; 15–25 min, 60% A; 25–32 min, 60–45% A; 32–35 min, 45–40% A; 35–38 min, 40–38% A; 38–40 min, 38–33% A; 40–45 min, 33–0% A.

### Cell viability of BELNs on RAW 264.7 cells by CCK8 assay

RAW 264.7 macrophages (RRID: CVCL_0462, derived from mononuclear macrophages of the BALB/c mouse strain and established via leukemia virus-induced transformation) were obtained from the Cell Bank of the Chinese Academy of Sciences on Oct 1, 2022 (order number: 241363) and confirmed to be contamination free. RAW 264.7 macrophages were cultured in complete medium composed of RPMI 1640 medium (Gibco, Cat. No. 11875093) supplemented with 10% FBS (Gibco, Cat. No. 20370106) and 1% penicillin‒streptomycin (Gibco, Cat. No. 15140122; 100 U/mL penicillin, 100 µg/mL streptomycin) and placed in a 5% CO_2_ cell incubator at 37 °C. The cells were seeded into a 96-well plate at a cell density of 1 × 10^5^ cells/mL and cultured at 37 °C for 24 h. Subsequently, cells were incubated with different gradient concentrations of BELNs (protein concentration, 0, 0.5, 2, 4, 8, 10 µg/mL) for 24 h. After washing with PBS buffer for 3 times, cells were incubated with CCK-8 solution (0.5 mg/mL) for 1 h, and the absorbance at 450 nm was measured. The cell viability of the blank control group is counted as 100%.

### In vitro anti-inflammatory activity of BELNs

RAW 264.7 macrophages were seeded into a 24-well plate at a density of 1 × 10^5^ cells/well and incubated with BELNs (protein concentration, 0, 2, and 8 μg/mL) for 24 h. Subsequently, the cells were co-incubated with basal medium containing LPS (1 μg/mL) for 4 h. The cells were harvested and centrifuged, and the concentrations of cellular inflammatory factors (TNF-α and IL-10) in the supernatants were quantified using ELISA kits.

### Uptake of BELNs by RAW 264.7 macrophages

BELNs were labeled with PKH26 for cell experiments. A 4 μL of PKH26 solution was diluted in 1 mL of Diluent C, mixed with BELNs for 2 min, and then added 2 mL 1% BSA solution to stop reaction. The mixtures were centrifuged at 100,000 × *g* at 4 °C for 1 h to remove free dyes. The precipitations were resuspended in PBS at the final concentration of 8 μg/mL. A dye solution mixed with PBS solution in the absence of BELNs was set as the negative control.

RAW 264.7 macrophages were seeded into a 12-well plate at a density of 5 × 10^4^ cells/well and incubated with PKH26-labeled BELNs (protein concentration, 8 μg/mL). After being incubated for 5 h, the cells were washed with PBS 3 times and fixed in paraformaldehyde solution (4%) for 20 min. The cells were then stained with DAPI at 37 °C for 30 min. Finally, the cells were observed and imaged using a fluorescence microscope (Nikon, Japan). To quantify the uptake efficiency of the BELNs, the cells were suspended in PBS and analyzed using a flow cytometer (BD Biosciences, USA) on the basis of 10,000 gated cell events.

To verify the glycoprotein receptor-mediated endocytosis of BLENs, RAW 264.7 macrophages were seeded in 6-well plates at a density of 2 × 10^5^ cells/well. After overnight incubation, cells were incubated with PKH26-loaded BELNs suspensions with or without galactose (200 µg/mL) for 3 h. Cells were washed with PBS, detached by using a sterile cell scraper, collected by centrifugation, and resuspended in PBS. The endocytosis of BELNs by RAW 264.7 macrophages with galactose was analyzed by fluorescence microscope and flow cytometry.

### In vivo distribution of BELNs

BELNs were labeled with DiR for in vivo bio-distribution. A DiR solution (5 mM) was mixed with BELNs, and the obtained mixture was incubated for 30 min at 37 °C. The labeled BELNs were centrifuged at 100,000 × *g* at 4 °C for 2 h to remove free dyes. Then the labeled samples were resuspended in PBS at a final concentration of 10 mg/mL. A dye solution mixed with PBS solution in the absence of BELNs was set as the negative control.

C57BL/6 mice were fasted for 12 h with free access to water. Each mouse was intragastrically administered 100 μL of DiR-labeled BELNs (10 mg/mL). The hair on the abdomen of the mice was shaved, and the in vivo fluorescence signal distribution after the administration of BELNs at 0, 1, 3, 5, and 7 h was observed and photographed using an IVIS Lumina small animal in vivo imaging system (PerkinElmer, USA). At the end of the investigation, the mice were euthanized by carbon dioxide inhalation. The heart, liver, spleen, kidneys, stomach, small intestine, and colon of the mice were removed using dissection equipment for ex vivo imaging. The fluorescence parameters of DiR were Ex = 748 nm and Em = 789 nm.

### Mice

C57BL/6 male mice (8 weeks of age) were obtained from Charles River Laboratories (Beijing, China). All animal experiments in this study were approved by the Experimental Animal Ethics Committee of Zhejiang University Institutional Animal Care and Use Committee (Approval No. 25069). The experiments were conducted in strict accordance with the ARRIVE Guidelines and the Guide for the Care and Use of Laboratory Animals (NIH Publication No. 85-23, revised 2011). The mice were housed at 22 ± 2 °C and 55 ± 5% humidity with a 12 h light/dark cycle and provided with a sterile standard chow diet.

### Treatment of DSS-induced IBD mice with BELNs

After a 1-week acclimatization period, 30 mice were randomly divided into three groups (*n* = 10 per group): water control, DSS model, and DSS + BELNs. In the DSS+BELNs group, BELNs (1 × 10^9^ particles/kg bw) were suspended in 200 µL of PBS and administered via oral gavage once daily for 14 days. Treatment was initiated concurrently with DSS treatment. The mice were fasted for 4 h with free access to water prior to oral administration of the BELNs. The mice in the DSS+BELNs group were orally administered BELNs (1 × 10^9^ particles/kg bw) once every day for 2 weeks throughout the experiment. As controls, the remaining two groups received an equivalent volume of PBS via oral gavage. Starting on Day 9, the mice in the DSS model group and the DSS + BELNs group drank freshly prepared 3.5% DSS-containing water. Mouse body weight, feces, and physical activity were monitored daily. On Day 16, the mice were euthanized, and serum was harvested for ELISA analysis. Various segments of intestinal tissues were harvested for histological analysis, RT‒qPCR, or Western blot analysis. For the phenotypic assessments (body weight, DAI, and colon length), 10 mice per group were used (*n* = 10). For molecular analyses, including Western blot and sequencing, to ensure adequate sample yield and minimize individual variation, samples from 2 to 3 randomly selected mice in the same group were pooled into one biological replicate, resulting in 3 to 5 pooled replicates per group.

### ELISA

Mouse serum was harvested by the orbital sinus method. The serum levels of inflammatory factors (TNF-α, IL-1β, and IL-10) were quantified by the corresponding commercial ELISA kits according to the manufacturer’s protocols.

### H&E staining

The different intestinal segments of mice (colon, jejunum, ileum, and duodenum) were collected and fixed in paraformaldehyde solution (4%, v/v) overnight. These tissues were dehydrated through an ethanol series, cleared in xylene, and embedded in paraffin. Sections (5 μm thick) were prepared using a microtome, and then dewaxed, rehydrated through a graded ethanol series, stained with hematoxylin and eosin (H&E), dehydrated again, cleared in xylene, and finally mounted. The intestinal histomorphology, including the integrity of the mucosal structure and inflammatory cell infiltration were observed using an optical microscope.

### Immunohistochemistry (IHC)

Paraffin-embedded colon tissues were prepared and stained according to the instructions of the immunohistochemistry kit. The tissue sections were respectively incubated with rabbit anti-MPO polyclonal antibody (1:200), rabbit anti-ZO-1 polyclonal antibody (1:200), and rabbit anti-MUC2 polyclonal antibody (1:200) for 1 h, followed by incubation with a second antibody (1:400) for 15 min. Nuclei were counterstained with DAPI, and the sections were mounted with glycerol-based mounting medium. The images were observed by microscopy, and the intensity arbitrary unit (a.u.) was measured by Image J software to evaluate the levels of oxidative stress and physical barrier-related proteins in colon tissues.

### RT-qPCR analysis

The intestinal tissues were sufficiently ground with Trizol reagent in a tissue grinder and lysed on ice for 15 min. The supernatant after centrifugation was successively mixed with chloroform and isopropanol to extract the total RNA. RNA samples that have undergone quantification and purity testing were used for reverse transcription reactions using the HiScript Ⅲ RT SuperMix for qPCR (+gDNA wiper) kit to obtain cDNA templates. qPCR reactions were performed according to the instructions of the Taq Pro Universal SYBR qPCR Master Mix kit in a CFX96 touch real-time qPCR detection system (Bio-Rad Laboratories, USA). The primer sequences of genes in this study are listed in Table S1.

### Western blotting

The intestinal tissues were sufficiently ground with RIPA reagent containing protease inhibitor cocktail in a tissue grinder and lysed on ice for 30 min. Total protein extraction solution was quantified using the BCA protein assay kit and pretreated with loading buffer. After SDS-PAGE electrophoresis and transfer to a PVDF membrane, primary antibody and secondary antibody were added for incubation. Finally, the images were acquired in a fully automatic chemiluminescence image analysis system.

### Gut microbiota analysis

On Day 16, fecal samples were collected from each mouse prior to euthanasia. To minimize environmental contamination, the mice were handled with sterile gloves, and pellets were obtained via gentle abdominal massage directly into sterile DNA-free tubes. For 16S rDNA sequencing, fecal samples from 2 to 3 mice within the same group were randomly pooled to form a single biological replicate (approximately 200 mg), resulting in *n* = 5 pooled replicates per group. Immediately after collection, the samples were snap-frozen in liquid nitrogen and stored at −80 °C for subsequent analysis. 16S rDNA sequencing analysis was performed at LC-Bio Technology Co., Ltd. (Shanghai, China). First, whole-genome DNA was extracted from the samples using a PowerFecal Pro DNA Kit (Qiagen). The V3–V4 hypervariable regions were amplified using broadly conserved primers, including 338F (5’-ACTCCTACGGGAGGCAGCAG-3’) and 806R (5’-GGACTACHVGGGTWTCTAAT-3’). PCR was performed in a 25 μL reaction mixture containing 12.5 μL of 2× Phanta Max Master Mix, 1 μL of each primer (10 μM), and 20 ng of template DNA. The thermal cycling conditions were as follows: initial denaturation at 95 °C for 3 min, followed by 27 cycles of denaturation at 95 °C for 30 s, annealing at 55 °C for 30 s, and extension at 72 °C for 45 s, with a final extension at 72 °C for 10 min. The PCR amplification products were subsequently purified using an AxyPrep DNA Gel Extraction Kit (Axygen Biosciences), quantified using a QuantiFluor^TM^ fluorometer (Thermo Fisher Scientific), and connected to sequencing adapters to construct a sequencing library. Purified amplicons were pooled in equimolar amounts and sequenced on the Illumina MiSeq PE300 platform (Illumina, San Diego) according to standard protocols.

Raw sequences were processed and quality-filtered using the QIIME2 (v2022.2) pipeline. Denoising was performed using DADA2 to obtain amplicon sequence variants (ASVs). OTUs were clustered at 97% similarity using UPARSE. Taxonomy was assigned to each OTU using the SILVA (release 138) database. Alpha diversity (Chao1, Shannon, Simpson, and Pielous_e) was calculated to assess microbial richness and evenness, and differences were analyzed via Kruskal‒Wallis tests. Beta diversity was visualized using principal coordinate analysis (PCoA) based on Bray‒Curtis distances and tested for significance using PERMANOVA.

To predict the functional potential of the gut microbiota, we performed PICRUSt2 (v2.3.0-b) analysis. The predicted functional profiles were categorized according to the Kyoto Encyclopedia of Genes and Genomes (KEGG) database at levels 1–3. Statistically significant differences in metabolic pathways between groups were determined using STAMP software (v2.1.3) with Welch’s t test.

### Statistical analysis

The results are presented as the mean ± standard deviation. Statistical analysis was performed using IBM SPSS Statistics 20 (USA) and GraphPad Prism 9.5 (USA). Differences between multiple groups were analyzed using one-way analysis of variance followed by Tukey’s post hoc test. For microbiota analysis (alpha diversity), the Kruskal‒Wallis test was used. A *p*-value < 0.05 was considered to indicate statistical significance.

## Supplementary information

Supplementary Materials

Supplementary Data 1

Supplementary Data 2

Supplementary Data 3

Supplementary Data 4

## Data Availability

The datasets generated and/or analyzed during the current study are not publicly available due to the ongoing further analysis of the comprehensive multi-omics (lipidomic, proteomic, and miRNA) profiles and potential patent applications related to BELNs processing, but are available from the corresponding author on reasonable request.
