# Supplementary material for: Chinese bayberry exosome-like nanoparticles attenuate DSS-induced colitis via immunomodulation, barrier restoration, and microbiota remodeling
Source: NPJ Sci Food. 2026 May 14;10:234. doi: 10.1038/s41538-026-00880-x (PMC13421505; doi:10.1038/s41538-026-00880-x)
Supplement: Supplementary file 1 — Supplementary Materials [file 41538_2026_880_MOESM1_ESM.pdf]

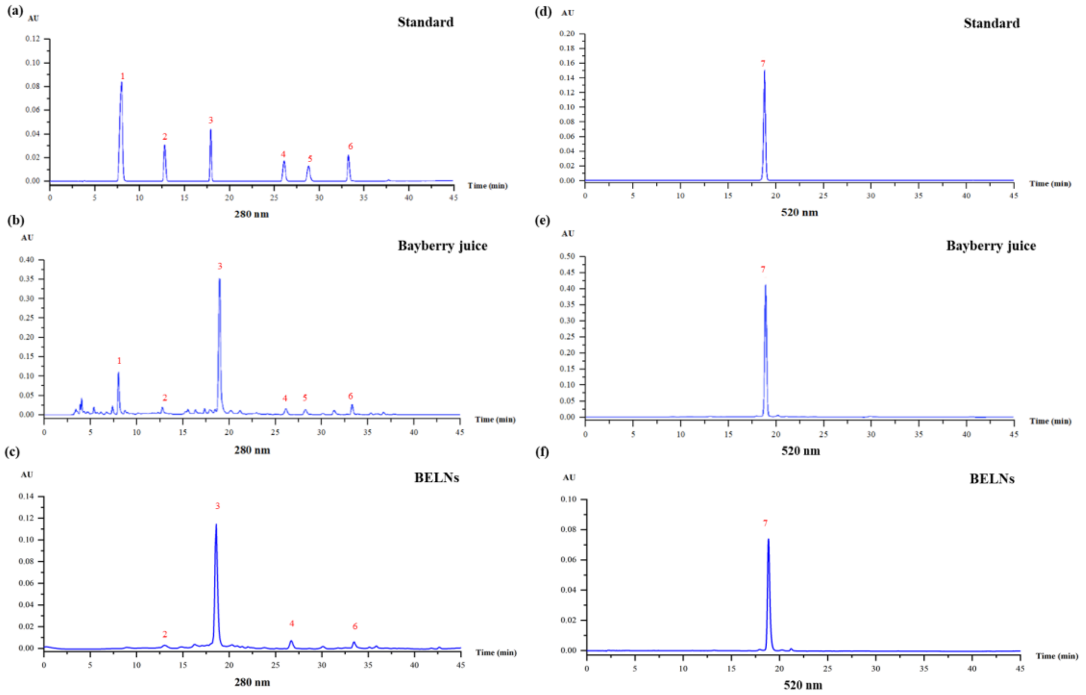

Fig. S1. HPLC curves of (a) different polyphenols standards, (b) bayberry juice, (c) BELNs at 280 nm. HPLC curves of (d) different polyphenols standards, (e) bayberry juice, (f) BELNs at 560 nm. Note: 1: gallic acid, 2: protocatechuic acid, 3: p-hydroxybenzoic acid, 4: myricitrin; 5: quercetin-3-O-glucoside, 6: quercitrin, 7: cyanidin-3-O-glucoside

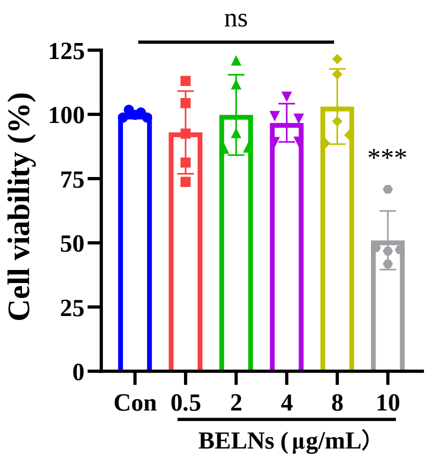

Fig. S2. Cell viability of BELNs on Raw 264.7 cells (n=5).

ns represented not significance, *p****<0.001.

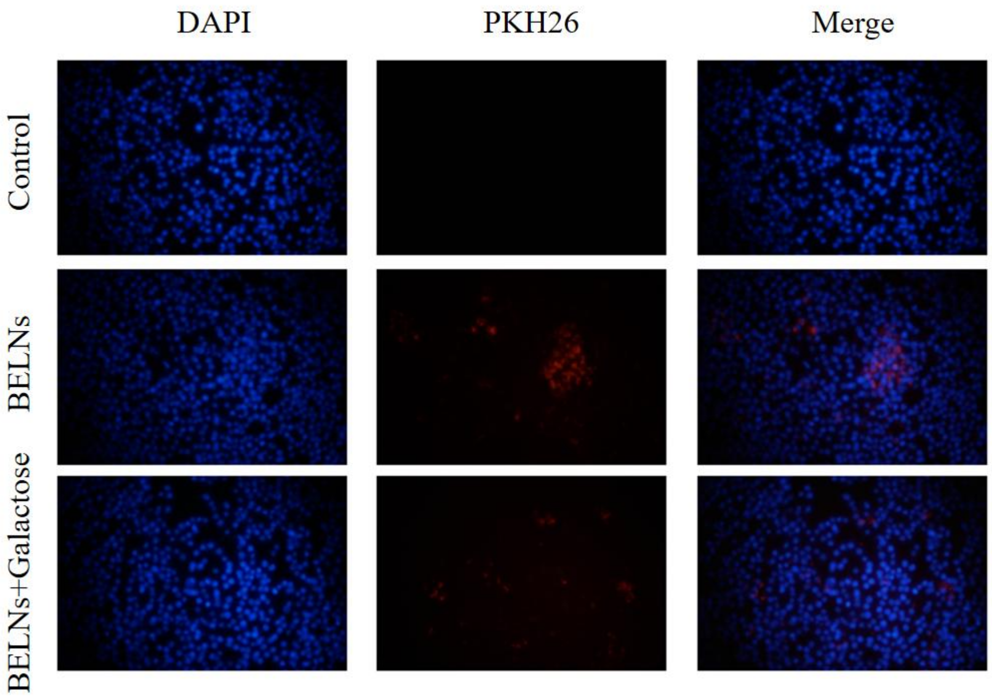

Fig. S3. The phagocytosis results of BELNs and galactose after co-cultured with Raw 264.7 macrophages for 5 h. The scale is 20 μm.

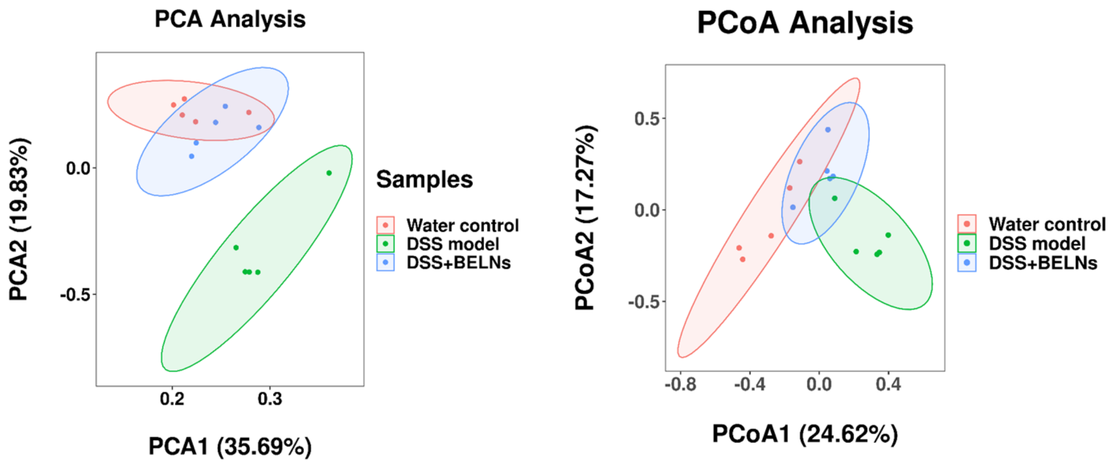

Fig. S4. β-diversity of fecal samples in different groups

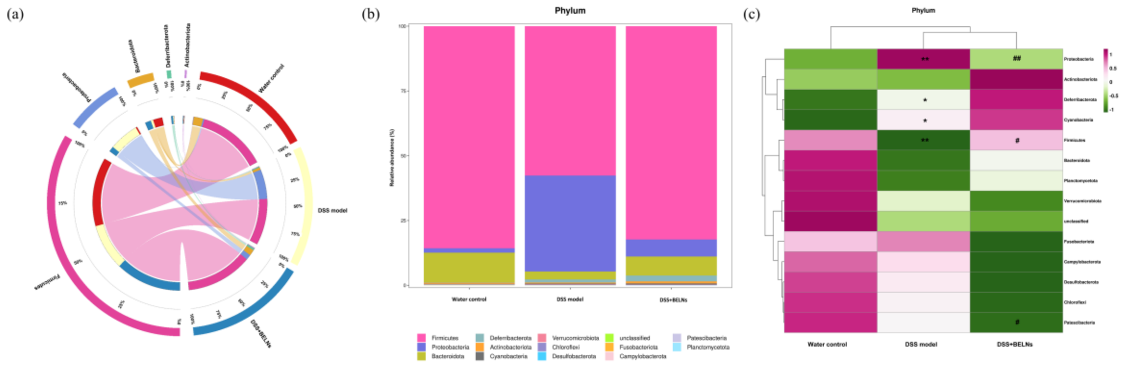

Fig. S5. BELNs treatments modulate gut microbiota in DSS-induced colitis mice.

(a) Circos plot of the top 5 dominant microbial groups (phylum level). (b) Gut microbiota composition and distribution (phylum level). (c) A phylum-level community heatmap. Data are pooled in one independent experiment with n=5 mice per group.

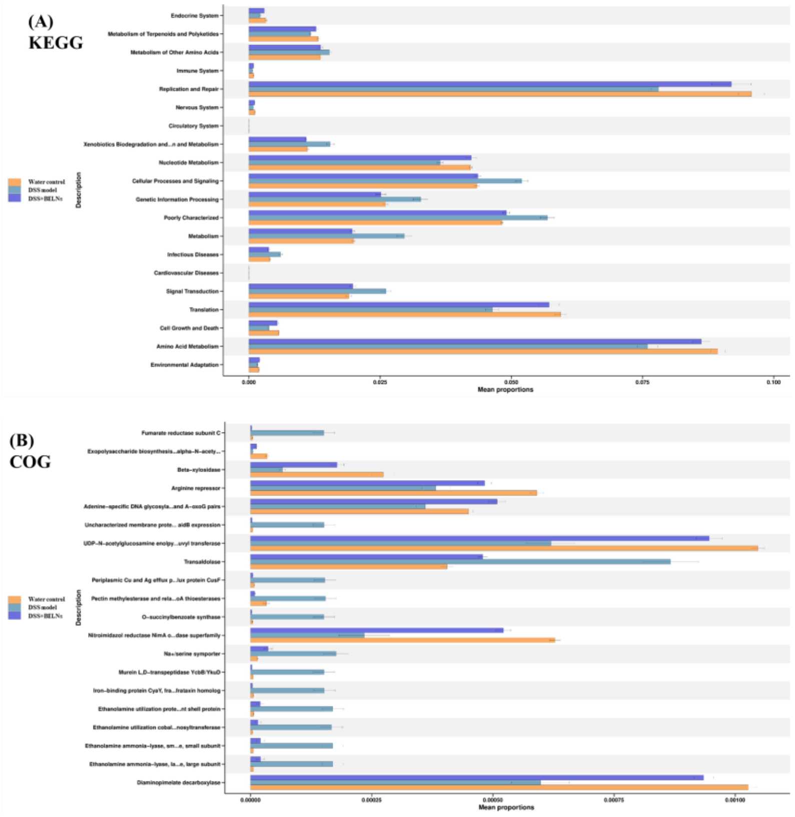

Fig. S6. PICRUSt2-based functional genomic prediction of the gut microbiota.

(a) Bar plot showing the mean proportions of predicted KEGG pathways (Level 3) across the Water control, DSS model, and DSS+BELNs groups. (b) Bar plot illustrating the predicted abundance of COG (Clusters of Orthologous Groups) functional categories among the three groups. Data are pooled in one independent experiment with n=5 mice per group.

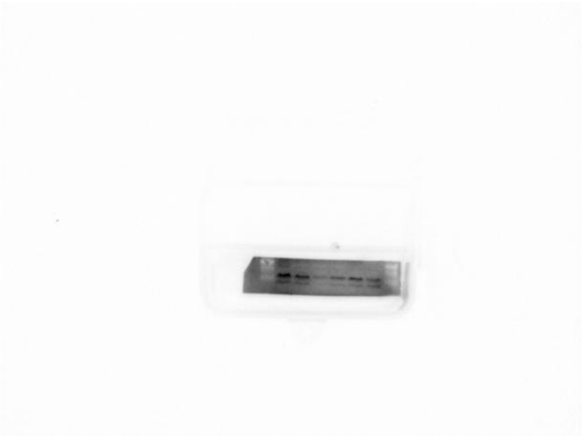

Fig. S7. Original western-blot image of IL-10

(left→right: con1, con2, DSS1, DSS2, DSS+BELNs1, DSS+BELNs2)

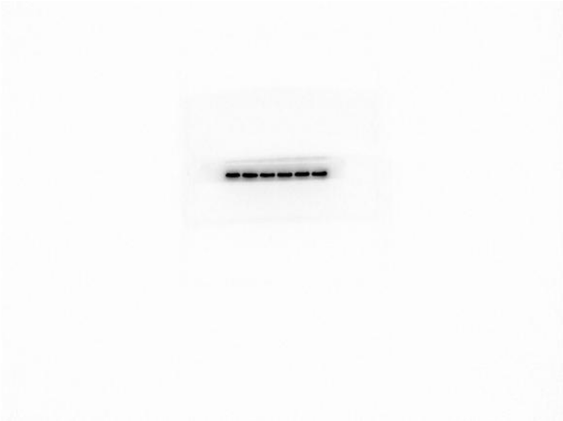

Fig. S8. Original western-blot image of GAPDH

(left→right: con1, con2, DSS1, DSS2, DSS+BELNs1, DSS+BELNs2)

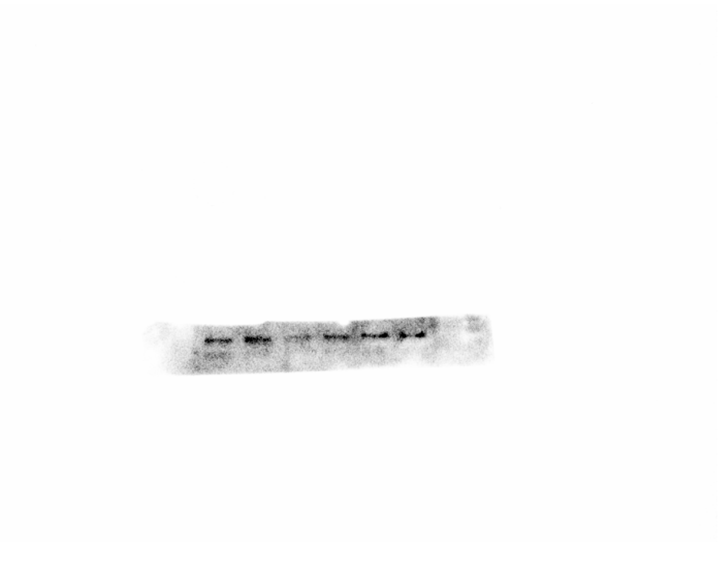

Fig. S9. Original western-blot image of IL-10

(left→right: con3, con4, DSS3, DSS4, DSS+BELNs3, DSS+BELNs4)

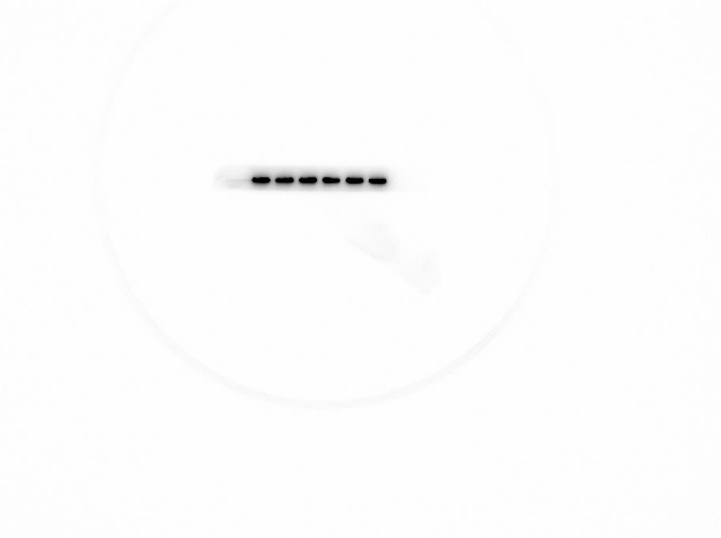

Fig. S10. Original western-blot image of GAPDH

(left→right: con3, con4, DSS3, DSS4, DSS+BELNs3, DSS+BELNs4)

Table S1. Primer sequences of genes in this study

Genes	Primers
GAPDH	F: ACCCCAGCAAGGACACTGAGCAAG
	R: GGCCCCTCCTGTTATTATGGGGGT
IL-10	F: TTTGAATTCCCTGGGTGAGAA
	R: GGAGAAATCGATGACAGCGC
TNF-α	F: AGCAGGCCATCACCACCAAGA
	R: GTGCGTCACATCCTTGAAGTCAT
IL-1β	F: TCGCAGCAGCACATCAACAAGAG
	R: TGCTCATGTCCTCATCCTGGAAGG

Supplementary Data 1. Proteomic analysis of BELNs by LC-MS/MS.

This Excel file provides the comprehensive dataset of proteins identified in BELNs. The dataset includes protein accession numbers, descriptions, molecular weights, and corresponding functional annotations based on the Gene Ontology (GO) database.

Supplementary Data 2. Lipidomic analysis of BELNs by LC-MS/MS.

This Excel file provides the comprehensive lipidomic profile of BELNs. It includes the identification of individual lipid species categorized by their class, along with their relative abundances, mass-to-charge ratios (m/z), and retention times.

Supplementary Data 3. miRNA sequencing analysis of BELNs.

This dataset presents the complete list of microRNAs (miRNAs) identified in BELNs *via* small RNA sequencing. It includes miRNA indices, names, exact sequences, species of origin (e.g., Morella rubra), predicted secondary structures (hairpins), GC content, and minimum free energy (dG) values for the identification of conserved and novel plant miRNAs.

Supplementary Data 4. PICRUSt2 KEGG analysis.

This file presents the PICRUSt2-based functional prediction of the gut microbiota, categorized according to the Kyoto Encyclopedia of Genes and Genomes (KEGG) database. It includes the relative proportions of functional pathways across the Water control, DSS model, and DSS+BELNs groups, with corresponding statistical significance levels.
